# Spatiotemporal distribution and determinants of measles incidence during a large outbreak, Italy, September 2016 to July 2018

**DOI:** 10.2807/1560-7917.ES.2019.24.17.1800679

**Published:** 2019-04-25

**Authors:** Xanthi D Andrianou, Martina Del Manso, Antonino Bella, Maria Fenicia Vescio, Melissa Baggieri, Maria Cristina Rota, Patrizio Pezzotti, Antonietta Filia

**Affiliations:** 1 European Programme for Intervention Epidemiology Training (EPIET), European Centre for Disease Prevention and Control (ECDC), Stockholm, Sweden; 2 Department of Infectious Diseases, National Institute of Health (Istituto Superiore di Sanità), Rome, Italy

**Keywords:** measles, surveillance, vaccination coverage, outbreak, Italy

## Abstract

**Background:**

Measles is still endemic in Italy and outbreaks are frequent. From 2016 to 2018, more than 7,000 measles cases were reported to the national integrated measles and rubella surveillance system, the largest outbreak since implementation of this system.

**Aim:**

We aimed to describe the characteristics and spatiotemporal distribution of measles cases in Italy and explore determinants of incidence at municipality level.

**Methods:**

We performed a retrospective observational study, mapping by municipality****all measles cases reported to the national surveillance system with symptom onset between 1 September 2016 and 31 July 2018. We also analysed measles–mumps-rubella (MMR) vaccination coverage (VC) data (2000–2017) for the first and second dose, collected from the Ministry of Health. We used regression analysis to explore factors associated with measles incidence at municipality level.

**Results:**

We analysed 7,854 cases, 3,927 (50%) female. Median age was 26 years; 475 cases (6%) were younger than 1 year. The outbreak occurred in two epidemic waves. The first started in central/northern regions (end of 2016), the second (mostly within 2018) was concentrated in southern regions. In 2016 and 2017, national VC was below 95% for both MMR doses. In 2017, only one region reported VC above 95% for the first dose. At municipality level, incidence was associated with higher urbanisation, less deprivation and fewer adults.

**Conclusion:**

The spread of measles between September 2016 and July 2018 in Italy indicates the need to improve VC and to explore further how societal and other parameters might be linked to incidence.

## Background

Measles is a highly infectious vaccine-preventable disease that can lead to severe complications and even death [1]. Widespread vaccination against measles has prevented an estimated 21.1 million deaths worldwide between 2000 and 2017 [1]. The disease has been targeted for elimination in all six World Health Organization (WHO) Regions [1] and one of the aims of the Global Vaccine Action Plan 2015–2020 is to eliminate measles in at least five regions by 2020. To date, the Region of the Americas is the only region to have reached this goal [2]. However, endemic transmission was re-established in Venezuela in 2018 [3]. In the WHO European Region, all countries have committed to the goal of eliminating measles and, according to the Regional Verification Commission for Measles and Rubella Elimination, 43 of 53 member states had in 2017 interrupted endemic transmission for at least 12 months; Italy was among the 10 countries where endemic transmission was still ongoing [4,5].

In Italy, the monovalent measles vaccine was introduced in 1976 and has been recommended by the Ministry of Health (MoH) since 1979 (one dose) [6,7]. The combined measles-mumps-rubella (MMR) vaccine was introduced in the late 1980s but vaccination coverage (VC) remained very low for many years: up to 1988, national VC in 2-year-old children was below 21%, in 1989 it was 41% and since then it has slowly increased [8]. In 2003, the first national measles elimination plan was implemented and a two-dose schedule was introduced, starting in the 2002 birth cohort, with the first dose given at 12–15 months of age and the second dose at 5–6 years [7]. The elimination plan was updated in 2010. In 2017, when MMR vaccination became mandatory for children up to the age of 16 years, VC was 91.8% for the first dose at age 2 years [9].

In Italy, measles has been a statutory notifiable disease since 1934 [6,10]. An enhanced national measles surveillance system was introduced in 2007, requiring all physicians to report suspected cases within 12 hours and local health authorities to conduct contact tracing and provide post-exposure prophylaxis to susceptible contacts. In 2013, measles and rubella surveillance were integrated to strengthen the surveillance of both diseases [11]. Suspected cases are reported through an online platform that collects individual case details, including age, date and place of onset of symptoms, vaccination status and outcome [11]. Cases are classified according to a standard case definition [12].

Measles incidence in Italy gradually decreased after the introduction of vaccination, however, large outbreaks continue to occur regularly [13,14]. Following a period of low incidence in 2015 (0.4 per 1,000,000 [15]) and in the first half of 2016 (0.7 per 1,000,000 in January–June 2016 [16]), an increase in the reported number of cases was observed in September 2016 and a large outbreak occurred in 2017 and 2018 [17].

The aim of the present study was to analyse measles cases reported in Italy during the outbreak, to describe their spatiotemporal distribution and evaluate possible determinants of measles incidence at municipality level.

## Methods

Italy is divided into 21 regions and autonomous provinces, thereafter referred to as ‘regions’ (Supplementary Figure S1) and 7,998 municipalities based on 2016 data from the Italian National Institute of Statistics (Istituto Nationale di Statistica; ISTAT) [18]. All regions participate in measles surveillance by reporting cases to the national integrated measles and rubella surveillance system.

In this retrospective observational study, we analysed reported measles cases with symptom onset between September 2016 and July 2018. Cases were classified as possible, probable or confirmed based on the standard European Union (EU) case definition [12], and as endemic (with known or unknown link to another endemic case), imported (exposure outside Italy 7–18 days before symptom onset), import-related or of unknown source based on their origin [19]. Administrative measles VC data, routinely collected by each region for the ages 2 and 5–6 years and reported to the MoH, were obtained from the MoH website [9,20].

Additional data used in this study were data at municipality level, including the degree of urbanisation, the social deprivation index (SDI), and the percentage of adults in each municipality. The degree of urbanisation was obtained from Eurostat data for 2016 [21]. The SDI of each Italian municipality was previously calculated by other authors in 2016, using variables on educational level (percentage of population having completed elementary school), percentage of unemployed population, percentage of houses that are rented, percentage of single-parent families and number of household occupants per 100 m^2^, obtained from the 2011 national census [22,23]. The percentage of adults (18 years and older) in each municipality was derived using the 2017 population estimates from ISTAT [24].

### Statistical analysis

A descriptive analysis of cases was performed for the complete study period and for each of the two epidemic waves September 2016 to November 2017 and December 2017 to July 2018. At the national level, weekly incidence rates were calculated and plotted. The log-transformed countrywide weekly incidence rates were smoothed using the locally weighted regression method (LOESS) of the R package ggplot2 [25]. At the regional level, we calculated the crude and age-adjusted incidence rates (direct standardisation using the Italian population as a reference, per 1,000,000 per year) for the whole study period and by month. Population data for 2017 retrieved from ISTAT were used in all calculations [26]. All incidence rates were calculated for the total population and by age group (< 1, 1–4, 5–19 and > 19 years).

For the spatiotemporal description of the outbreak, cases were aggregated by month and municipality of onset using as reference the maps available by ISTAT [18]. If information on municipality of onset was not available (1,022 cases), municipality of residence, municipality in which the case was registered or municipality of notification were used as a proxy (in that order). When all of the aforementioned variables were missing (142 cases) but the region of notification was known, we imputed the values for the municipality of onset using the multivariate imputation by chained equations method and taking into consideration age and sex. The values used were those of the last of five datasets all generated after five iterations [27]. Details on the cases for which the municipality of onset was imputed are available in Supplementary Figure S2. Crude and cumulative incidence rates per 1,000,000 per year were calculated by municipality and mapped by month. In the maps, the incidence was categorised using the quintiles of the distribution of all incidence values greater than zero.

Summary statistics of VC at 2 years of age (first dose) for the years 2000 to 2017 by region were calculated. VC data between 2013 and 2017 were mapped by region and year, for those aged 2 years (first dose) and 5–6 years (two doses).

Regression analysis was conducted to identify explanatory parameters of incidence at municipality level. We used three explanatory variables: (i) urbanisation level (categories: densely populated, intermediate and thinly populated areas), (ii) SDI (categories from 1 to 5, referring to increasing levels of deprivation) and (iii) percentage of adults (≥ 18 years-old) in the municipality, categorised according to the quartile values of the distribution in the following categories: (73.14%–82.76%, 82.77%–84.64%, 84.65%–86.65% and > 86.65%). The variables urbanisation and SDI were selected based on previous literature findings on links between measles and these two parameters, while the percentage of adults was used as an indicator of the population structure in each municipality, given the hypothesis that municipalities with more adults might be less susceptible because of past exposure of the adults to measles [28-30]. In the regression analysis, the incidence by municipality was modelled using negative binomial distribution, and incidence rate ratios (IRR) were calculated. Crude and region-adjusted (region as random effect) univariable and multivariable models were performed with each of the three variables separately and together. The regression models where SDI was a predictor were repeated in a set including only municipalities with populations smaller than 50,000 in a sensitivity analysis because of concerns that the SDI in municipalities with larger populations may not be representative of the overall social deprivation categories encountered in the same area [23].

Data analysis was conducted in R (version 3.5.3) using Rstudio (version 1.1.453) [31,32]. A reference list of all the packages used in the analysis, as well as the RECORD checklist for studies reporting routinely collected data for this analysis are available in Supplementary Tables S1 and S2. The scripts have also been made available in the Supplementary material.

## Ethical statement

This study was conducted using data from the Italian national integrated measles and rubella surveillance routinely collected and analysed within the mandate of the Italian National Institute of Health; therefore, no ethical approval was necessary.

## Results

### Characteristics and distribution of reported measles cases

From September 2016 to July 2018, 7,869 possible, probable and confirmed measles cases were reported to the national surveillance system, of which 15 were excluded from the present analysis because of incomplete information. Of the remaining 7,854 cases, 6,215 (79.1%) were laboratory confirmed and 6,969 (88.7%) were endemic (Table 1); 3,852 (49%) cases were hospitalised. The median age of cases was 26 years (range: 1 day–84 years), with 475 (6%) cases younger than 1 year and 279 (3.5%) cases older than 50 years. Table 1 shows the main characteristics of reported cases for the complete study period and for each of the two intervals that roughly define different periods of the outbreak (September 2016–November 2017 and December 2017–July 2018).

**Table 1:** Characteristics of measles cases reported to the national surveillance system, Italy, September 2016–July 2018 and in the two epidemic waves during this period (n = 7,854)

	Complete study period September 2016–July 2018		September 2016–November 2017 ( first wave)		December 2017–July 2018 (second wave)	
	N	%	n	%	n	%
Total cases	7,854		5,584		2,270	
Age group (years)						
< 1	475	6.0	330	5.9	145	6.4
1–4	980	12.5	688	12.3	292	12.9
5–19	1,093	13.9	793	14.2	300	13.2
> 19	5,306	67.6	3,773	67.6	1,533	67.5
Sex						
Female	3,927	50.0	2,856	51.1	1,071	47.2
Male	3,927	50.0	2,728	48.9	1,199	52.8
Classification						
Confirmed	6,215	79.1	4,486	80.3	1,729	76.2
Possible	1,083	13.8	662	11.9	421	18.5
Probable	556	7.1	436	7.8	120	5.3
Origin of infection						
Endemic	6,969	88.7	5,006	89.6	1,963	86.5
Import-related	72	0.9	44	0.8	28	1.2
Imported	103	1.3	62	1.1	41	1.8
Not indicated	710	9.0	472	8.5	238	10.5
Vaccination status						
Non-vaccinated	6,323	80.5	4,351	77.9	1,972	86.9
Vaccinated	795	10.1	609	10.9	186	8.2
Not specified	736	9.4	624	11.2	112	4.9
Number of doses (among those with status ‘vaccinated’)						
1	473	59.5	357	58.6	116	62.4
2	109	13.7	77	12.6	32	17.2
Not specified	213	26.8	175	28.7	38	20.4

**Figure 1:**
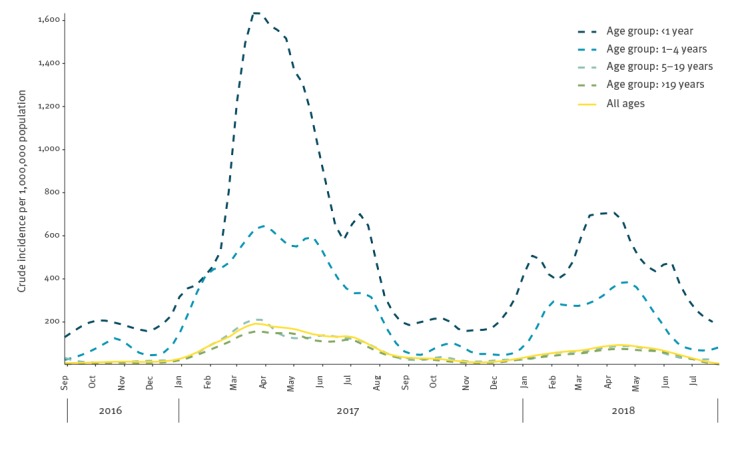
Weekly incidence (per 1,000,000 population) of reported (possible, probable and confirmed) measles cases, by age group and for the total study population, Italy, September 2016–July 2018 (n = 7,854)

Figure 1 shows the weekly incidence rate of measles in Italy in the period September 2016 to July 2018. Two waves were observed: a first increase in measles incidence occurred in September 2016 (n = 53 cases); the number of cases then increased sharply in January 2017 (n = 296) compared with 88 cases in December 2016. This first wave peaked in March 2017 (n = 973 cases) and the number of reported cases gradually decreased until November 2017 (n = 66 cases). The second wave started in December 2017 and peaked in April 2018 (n = 466 cases). Overall, in all time points of the study period, the incidence was higher in infants (< 1 year-old) than in other age groups. Crude incidence for the whole study period was 67.6 per 1,000,000 per year.

The lines have been smoothed using the local weighted regression method (LOESS).

Incidence varied widely between the regions and the highest annual age-adjusted incidence rate was observed in the Lazio region in central Italy (193 per 1,000,000). Most cases in Lazio were reported during the first wave, while during the second wave, most cases were reported from the southern regions (e.g. Sicily, Calabria). Sicily had the second highest overall incidence rate (149 per 1,000,000) during the study period. The standardised incidence rates for each region by month for the total study population and by age group are available in Supplementary Figure S3. With regards to the burden of measles by age group, more than 67% of cases were older than 19 years but the highest incidence was observed in the age group younger than 1 year (529.9 cases per 1,000,000), both nationally during the whole study period and in the regions that were most affected by the outbreak. In the Lazio region, which was mostly affected during the first wave, the incidence among infants was 1,550.2 per 1,000,000; in Sicily, which was mostly affected during the second wave, it was 1,316.5 per 1,000,000. The second highest incidence was observed in the age group 1–4 years (Supplementary Table S3).

**Figure 2:**
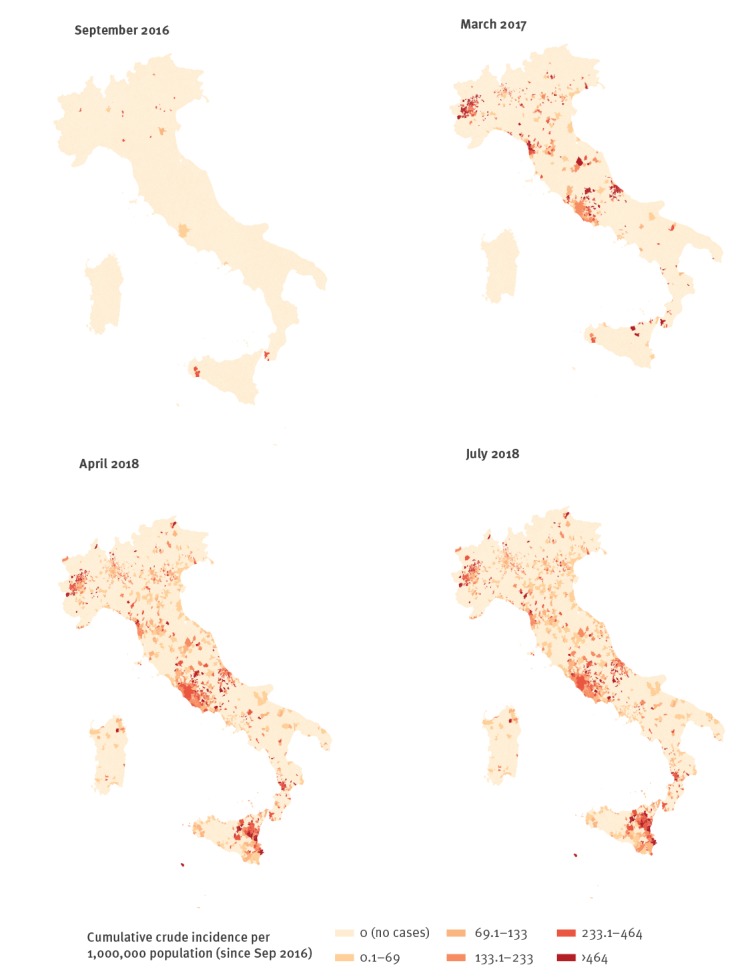
Cumulative measles incidence (per 1,000,000 per year) at beginning and end of the study period and snapshots of the cumulative incidence in March 2017 and April 2018, showing the spread of the cases in different municipalities, Italy, September 2016–July 2018 (n = 7,854)

At the municipality level, between September and October 2016, only a few municipalities in nine and 10 regions reported cases, 28 municipalities in September and 39 in October of the 7,998 municipalities analysed. The number of municipalities involved in the outbreak increased in central and northern Italy (first wave) in the subsequent months, with a peak in March 2017 when 373 (5%) municipalities in 18 regions reported at least one case. In November 2017, cases were reported by only 41 municipalities (66 cases in eight regions). In December 2017, a new increase was observed (second wave) with transmission occurring mostly in southern municipalities and in the region of Sicily. In April 2018 (peak of the second wave), 157 municipalities in 15 regions reported at least one case. Throughout the study period, in total 1,512 (19%) of the 7,998 municipalities reported at least one case (Figure 2).

Animations of the crude and cumulative (since September 2016) incidence of measles by month at municipality level are provided in the supplementary material (Videos S1 and S2).

### Determinants of measles incidence

#### National and regional vaccination coverage in Italy, 2000–2018

**Figure 3:**
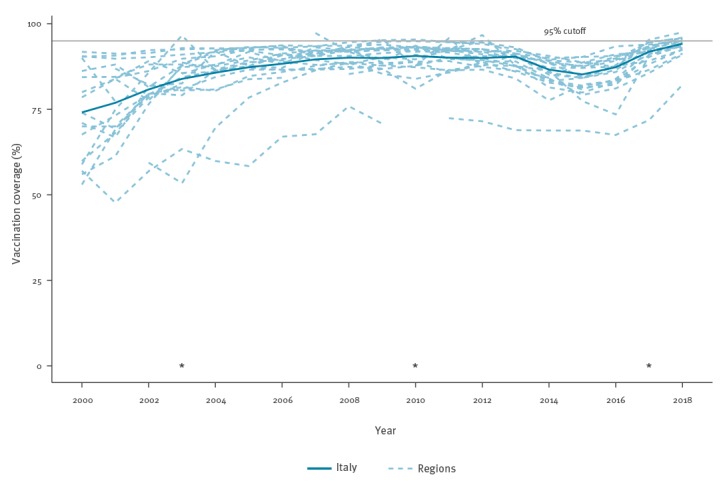
Vaccine coverage for the first dose of MMR vaccine at 2 years of age, in Italy and in each of the regions and autonomous provinces, 2000–2018

According to the annual reports published by the Italian MoH, the average regional VC for the first dose of measles-containing vaccine at 2 years of age increased between 2000 and 2008 from 74.1% to 90.1%. This increase was followed by a plateau until 2012 and a decrease after 2013 with a low point in 2015 at 85.3%. In 2016, the VC was still below 90%, at 87.3%, but in 2017 it reached 91.8%. Based on data released by the MoH in December 2018, the national VC for the first half of 2018 was 94.2%. In Figure 3, the major policy milestones towards measles elimination are noted along with the national and regional VC rates.

MMR: measles-mumps-rubella.

* Years when the two National Elimination Plans were implemented (2003 and 2010), and the year the mandatory vaccination was introduced (2017).

For 2018, the vaccination coverage refers to the first six months (until June 2018).

The average VC for the period 2000 to 2017, for the first dose of MMR vaccine measured at 2 years of age, differed among the 21 regions of Italy, ranging from a minimum of 65.6% for the Province of Bolzano to 91.8% in Emilia-Romagna (Supplementary Table S4). The maximum VC was 97.3% in Molise in 2005. Only in six regions was the VC higher than 95% during at least one of the 18 years (2000–2017) analysed (Supplementary Table S5). In 2017, only one region reported first-dose VC above 95% (Lazio region).

**Figure 4:**
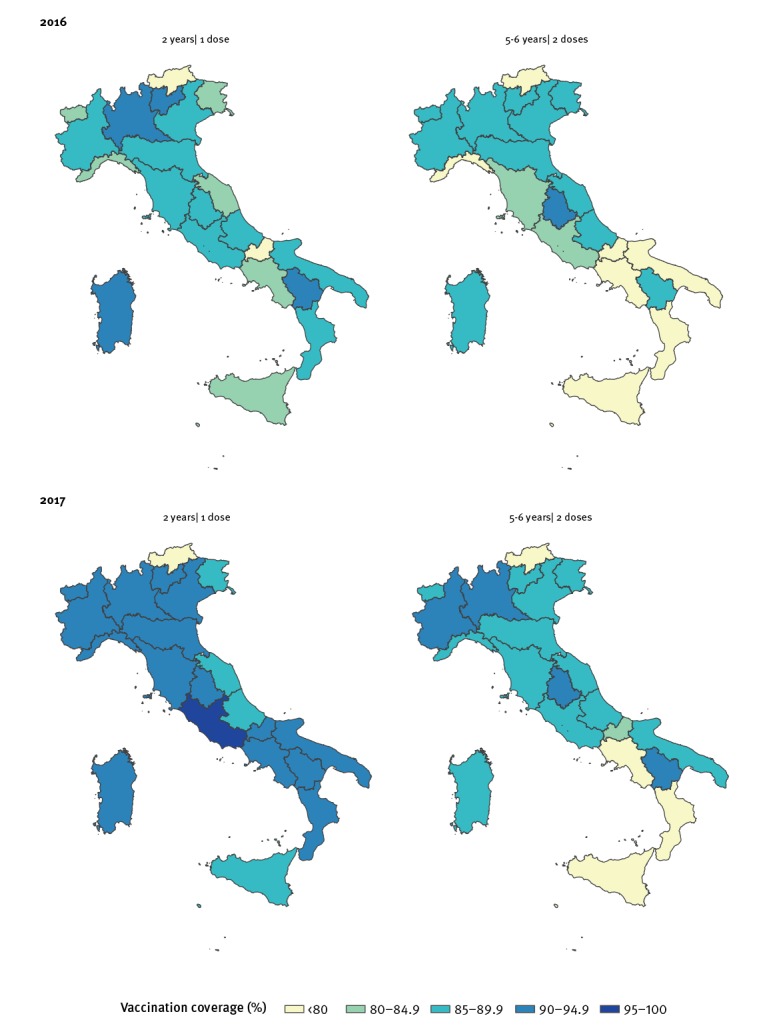
Vaccination coverage by year and region for the first dose of MMR vaccine at age 2 years and for the second dose at age 5–6 years, Italy, 2016–2017

VC for the second dose among 5–6-year-olds (available only since 2013) was consistently below 95% in all regions until 2017. Overall, most regions noted an increase in 2017 (Figure 4). Additional maps of the VC for the period 2013 to 2015 can be found in Supplementary Figure S4.

MMR: measles-mumps-rubella.

#### Municipality characteristics

Table 2 shows the IRR estimated for the characteristics of the municipalities explored in this analysis with negative binomial regression. In the univariable regression analyses adjusted for the region as a random effect, we saw a significantly higher incidence of measles in urban municipalities (densely populated areas), in municipalities with the lowest vs those with the highest deprivation levels and in municipalities in the quartiles with lower percentage of adults vs those in the upper quartile (> 86.65%). The association between urbanisation and incidence in the multivariable models, adjusting for the SDI and the percentage of adults, remained the same (Table 2). Adjusting for the urbanisation and the percentage of adults, the least deprived municipalities (SD 1) retained a higher incidence rate (IRR = 1.376; 95% CI: 1.033–1.834) than the municipalities with the highest deprivation (SD 5). In the multivariable model of the sensitivity analysis, where only the smaller municipalities (population < 50,000) were included, the previously observed trends (i.e. higher incidence in urban municipalities and those with higher SDI and fewer adults) were retained. Results of the univariable and multivariable models not adjusted for the regional effect as well as the sensitivity analysis model can be found in Supplementary Tables S6 and S7.

**Table 2:** Regression analysis results (univariable and multivariable models adjusted for the regional effects), measles outbreak, Italy, September 2016–July 2018 (n = 7,998 municipalities)

		Univariable models				Multivariable model			
		IRR	2.5% CI	97.5% CI	p value	IRR	2.5% CI	97.5% CI	p value
Degree of urbanisation Reference category: Thinly populated areas	Densely populated areas	1.929	1.546	2.407	<0.001	1.696	1.358	2.118	<0.001
	Intermediate	1.299	1.154	1.462	<0.001	1.158	1.022	1.311	0.021
Social deprivation index category Reference category: SD 5 (most deprived)	SD 1 (least deprived)	1.415	1.072	1.868	0.014	1.376	1.033	1.834	0.029
	SD 2	1.136	0.870	1.481	0.349	1.124	0.853	1.480	0.406
	SD 3	0.933	0.732	1.188	0.574	0.946	0.736	1.216	0.666
	SD 4	1.082	0.898	1.303	0.408	1.099	0.906	1.333	0.339
Percentage of adults (≥ 18 years-old) Reference category (> 86.65%):	73.14%–82.76%	1.970	1.590	2.442	<0.001	1.797	1.432	2.255	<0.001
	82.77%–84.64%	1.986	1.611	2.448	<0.001	1.858	1.495	2.310	<0.001
	84.65%–86.65%	1.629	1.318	2.013	<0.001	1.538	1.237	1.912	<0.001

CI: confidence interval; IRR: incidence rate ratios.

## Discussion

We have described case characteristics and spatiotemporal distribution of measles cases during the largest outbreak that has occurred in Italy since implementation of the national integrated measles and rubella surveillance system in 2013. The outbreak started in September 2016, affecting mainly central and northern regions, and spread in the following year to southern regions, showing a two-wave temporal pattern. All of Italy’s 21 regions and autonomous provinces reported at least one case during the study period, however, the absolute number of cases and the incidence rate were not equally distributed among them, as also observed in previous outbreaks in Italy [6,7,33].

The observed spatial differences in measles incidence were probably due to several factors. Overall, Italy has had very low measles VC for many years after vaccine introduction in the late 1970s and never reached the very high population immunity levels (95% two-dose coverage) required for measles elimination. National guidelines and recommendations on vaccination strategies are provided by the Italian MoH. However, the Italian health system is highly decentralised and immunisation activities are coordinated at the regional level. The various regions may therefore have implemented different strategies for promoting uptake, including catch-up programmes over the years. Wide differences in VC have been observed between regions throughout the years since vaccine introduction, with some regions reporting much lower coverage than others, leading to varying degrees of accumulation of susceptible population in Italian regions. Also, a certain degree of under-reporting, especially by primary care physicians, is still likely to be occurring, as reported by some previous studies; historically, this has been greater in southern regions than in northern regions [33-35]. The high number of hospitalisations observed during this outbreak may be associated with the fact that most cases were older than 20 years [36]. However, it also suggests that cases are being reported mostly by hospital physicians and less so by primary care physicians, as also described in other countries in Europe [37]. Different strategies for hospitalising measles patients may exist in the different Italian regions, which may have led to varying proportions of hospitalised cases and possibly also of notified cases between regions. However, analysing this was beyond the scope of our study. Studies should be performed to estimate possible variations in hospitalisation practices, the degree of under-reporting of measles in Italy and which measures should be taken to improve reporting by primary care physicians.

Our data indicate that the observed spatial differences may also be associated with the existence of large urban centres in the most affected regions and in differences in population distribution between the different regions. This could at least partially explain why the autonomous provinces of Trento and Bolzano, where there are no large urban centres, were less affected by the outbreak even if VC has also been suboptimal. However, other factors such as earlier outbreaks depleting the susceptible population may also have played a role. At the municipality level, measles incidence in this outbreak was significantly associated with greater urbanisation and with a lower percentage of adults in the population. Only 19% of the 7,998 municipalities analysed reported at least one measles case in the study period; however, these municipalities host over 35,000,000 population according to the 2017 population estimates, which is more than half of the total national population (estimated to be 60,589,445 in 2017 according to ISTAT). Previous studies outside Italy have also suggested that there is an uneven burden of measles between large and small cities [29]. Higher measles transmission in urban settings can be explained by higher population density and has previously been described in other countries such as Ireland, China and Ecuador [28,29,38]. With regards to higher incidence in municipalities with fewer adults, this may be due to the fact that measles transmission seems to be mainly driven by children [39].

Finally, we observed a higher incidence of measles in municipalities with less deprivation (lower SDI). This is an unexpected finding as we would expect that less deprivation would have a protective effect on health outcomes. Although measles outbreaks have been reported in socio-economically disadvantaged populations and/or areas [28,38,40], few studies have analysed the relation between socioeconomic factors and infectious diseases. A study in the Netherlands suggested that for various vaccine-preventable diseases, socioeconomic status is associated with antibody levels. [38]. More specifically, the authors found that people with lower socioeconomic status had higher antibody levels against some pathogens (e.g. pneumococcus, and meningococcus C) but lower IgG levels against measles. The latter is in agreement with our study. Also in agreement with our study, a study in Ecuador found a direct association between higher educational level of the head of the household and the greater occurrence of measles [38]. In Australia, a study in 2017 showed that immunisation gaps existed in areas with low and high socioeconomic status but the underlying reasons were different, possibly including, respectively, less access to healthcare and more concerns about vaccines [42]. Finally, the relation between individual socioeconomic status and occurrence of vaccine hesitancy also needs to be better understood. The literature is inconclusive about the exact association as social and other contextual factors are expressed differently in different communities, leading to different population health outcomes. In Italy, a study in 2018 did not find any association between employment or education and vaccination hesitancy at individual level [43].

Measles surveillance plays a fundamental role in measles elimination and has in the last 10 years been strengthened in Italy. Continuous monitoring of transmission is an ongoing activity at national, regional and local level. However, SDI and a possible connection with infectious diseases incidence has not previously been analysed in Italy, although this indicator is used in studies of non-communicable diseases [23]. Future studies can validate the use of SDI as an indicator, the only one currently available in Italy, or develop other indicators for the description of the effect of socioeconomic parameters in measles incidence.

Some limitations should be considered when interpreting the results of this study. These include lack of information on additional confounders to include in modelling at high spatial resolution. For example, VC at the level of the municipality and/or province was not available. Also, VC for adults was not systematically collected before 2016 and could not be considered. This prevented us from identifying whether observed differences in measles incidence by municipality or by province were a direct outcome of lower VC. Moreover, our study was ecological and the indicator we used for the SDI was derived from different municipality characteristics (including education, unemployment and housing aspects). This indicator was not developed specifically for our study but represents the best available data at the municipality level. Therefore, our results cannot be directly compared with other epidemiological studies on vaccinations in Italy, which used individual data on socioeconomic status (income, education etc). Another limitation is related to the fact that to calculate the incidence, we used the total population and not only the susceptible population (i.e. those unvaccinated and those not responding to the vaccine, who never had the disease). This may have led to underestimation of the true burden of measles among the susceptible individuals. Including factors that could lead to variations in the susceptible populations at subnational level such as previous outbreaks or differential birth rates, was beyond the scope of this study. 

Despite the above limitations, this is the first study that describes how measles cases in Italy were distributed at the local (municipality) level. The description and timely analysis of geographical distribution of measles transmission may be useful to public health authorities to identify areas of emerging transmission and areas where intensifying public health measures may be necessary to prevent and mitigate future outbreaks. Measures include closing existing immunity gaps and also improving contact tracing of cases, offering timely post-exposure prophylaxis (including MMR vaccination and use of immunoglobulins as appropriate) to susceptible contacts, and improving communication and the quality of vaccination services. Local MMR coverage data are crucial to plan targeted preventive actions, but also data on knowledge attitudes and practices in susceptible groups and possibly serosurveys of the population. The recent law making MMR vaccination mandatory in Italy will allow improving vaccination coverage up to the age of 16 years [44]. However, considering the median age of cases (26 years), elimination will also require supplementary vaccination activities targeted at older age groups. This is being addressed in an updated national elimination plan which is expected to be released in 2019. Improving communication and quality of vaccination services are other public health interventions that may need to be addressed. 

## Conclusions

The spread of measles between September 2016 and July 2018 in Italy indicates the need to improve VC and to explore further how societal and other parameters might be linked to incidence. The high median age of measles cases throughout the country highlights that immunity gaps need to be closed not only in children but also in the adult population. There is considerable variability among the Italian municipalities in terms of urbanisation, population structure and socioeconomic background, and we were able to identify trends between measles incidence and some municipality characteristics, e.g. higher incidence in urban centres and areas with fewer adults and higher incidence in areas with less social deprivation, highlighting the need to better understand the impact of socioeconomic factors on measles incidence. Future studies should account for additional confounders at local level, such as VC at the municipality level and broader socioeconomic characteristics. Further insight into this relationship may help inform policies to decrease measles incidence and achieve elimination.

## Supplementary Data

242700 SupplementaryMaterial_Figures-and-Tables

## Supplementary Data

449000 SupplementVideoS1_Incidence

## Supplementary Data

538000 SupplementVideoS2_CumulativeIncidence

## Supplementary Data

41000 SupplementScripts

## References

[1] World Health Organization (WHO). Measles. Geneva: WHO. [Accessed: 14 Apr 2019]. Available from: http://www.who.int/mediacentre/factsheets/fs286/en/

[2] Pan American Health Organization (PAHO)/World Health Organization (WHO). Measles elimination in the Americas. Washington: PAHO, Geneva: WHO. [Accessed: 8 Feb 2018]. Available from: http://www.paho.org/hq/index.php?option=com_content&view=article&id=12526%3Ameasles-elimination-in-the-americas&catid=6648%3Afact-sheets&Itemid=40721&lang=en

[3] Paniz-Mondolfi AE Tami A Grillet ME Márquez M Hernández-Villena J Escalona-Rodríguez MA Resurgence of Vaccine-Preventable Diseases in Venezuela as a Regional Public Health Threat in the Americas. Emerg Infect Dis. 2019;25(4):625-32. 10.3201/eid2504.181305 30698523 PMC6433037

[4] World Health Organization (WHO). Measles and rubella surveillance data. Geneva: WHO. [Accessed: 8 Feb 2018]. Available from: http://www.who.int/immunization/monitoring_surveillance/burden/vpd/surveillance_type/active/measles_monthlydata/en/

[5] World Health Organization Regional Office for Europe (WHO/Europe). Seventh meeting of the European Regional Verification Commission for Measles and Rubella Elimination (RVC). Copenhagen: WHO/Europe. [Accessed: 16 Oct 2018]. Available from: http://www.euro.who.int/__data/assets/pdf_file/0008/378926/7th-RVC-Meeting-Report-FINAL.pdf

[6] Filia A Tavilla A Bella A Magurano F Ansaldi F Chironna M Measles in Italy, July 2009 to September 2010. Euro Surveill. 2011;16(29):19925. 21801692

[7] Filia A Bella A Rota M Tavilla A Magurano F Baggieri M Analysis of national measles surveillance data in Italy from October 2010 to December 2011 and priorities for reaching the 2015 measles elimination goal. Euro Surveill. 2013;18(20):20480. 23725868

[8] Accordo tra il Ministro della salute, le regioni e le province autonome di Trento e Bolzano sul documento recante: «Piano nazionale per l’eliminazione del morbillo e della rosolia congenita». [Agreement between the Minister of Health, the regions and autonomous provinces of Trento and Bolzano on the document: “National plan for the elimination of measles and congenital rubella”].Rome: Ministero della Salute; 2003. Available from: http://www.trovanorme.salute.gov.it/norme/dettaglioAtto?id=9081&completo=true

[9] Vaccinazioni dell’età pediatrica e dell’adolescente. Coperture vaccinali. [Vaccinations at paediatric and adolescent age. Vaccination coverage]. Rome: Ministero della Salute. [Accessed: 13 Feb 2018]. Italian. Available from: http://www.salute.gov.it/portale/documentazione/p6_2_8_3_1.jsp?id=20

[10] Ciofi degli Atti M Filia A Revello MG Buffolano W Salmaso S Rubella control in Italy. Euro Surveill. 2004;9(4):19-21. 10.2807/esm.09.04.00462-en 15192262

[11] Reparto Epidemiologia delle Malattie Infettive ISS-CNESPS. Sorveglianza Integrata del Morbillo e della Rosolia. [Integrated surveillance of measles and rubella]. [Accessed: 13 mar 2018]. Italian. Available from: https://old.iss.it/site/RMI/morbillo/Default.aspx?ReturnUrl=%2fsite%2frmi%2fmorbillo%2f

[12] European Commission. Commission Implementing Decision of 8 August 2012 amending Decision 2002/253/EC laying down case definitions for reporting communicable diseases to the Community network under Decision No 2119/98/EC of the European Parliament and of the Council. Official Journal of the European Union. Luxembourg: Publications Office of the European Union; 2012:L262. Available from: http://data.europa.eu/eli/dec_impl/2012/506/oj/eng

[13] Grandolfo ME Medda E Novello F Ridolfi B Seroepidemiological evaluation of 1989-91 mass vaccination campaigns against measles, in Italy. Epidemiol Infect. 1998;121(3):645-52. 10.1017/S095026889800154X 10030715 PMC2809573

[14] EpiCentro. Morbillo. Aspetti epidemiologici. [Measles. Epidemiological aspects]. Rome: Istituto Superiore di Sanità. [Accessed: 16 Feb 2018]. Italian. Available from: http://www.epicentro.iss.it/problemi/morbillo/epidItalia.asp

[15] EpiCentro. Morbillo e rosolia News. Aggiornamento mensile gennaio 2016. [Measles and rubella news: monthly report January 2016]. Rome: Istituto Superiore di Sanita; 2016. Italian. Available from: http://www.epicentro.iss.it/morbillo/bollettino/RM_News_2015_23.pdf

[16] EpiCentro. Morbillo e rosolia News. Aggiornamento mensile luglio 2016. [Measles and rubella news: monthly report July 2016]. Rome: Istituto Superiore di Sanita; 2016 Italian. Available from: http://www.epicentro.iss.it/morbillo/bollettino/RM_News_2016_29.pdf

[17] EpiCentro. Morbillo e rosolia News: il bollettino della sorveglianza integrata morbillo-rosolia. [Measles and rubella news: the bulletin on measles-rubella integrated surveillance]. Rome: Istituto Superiore di Sanita. [Accessed: 4 Jul 2018]. Italian. Available from: https://www.epicentro.iss.it/morbillo/bollettino

[18] Istituto nazionale di statistica (ISTAT). Archivio dei confini delle unità amministrative a fini statistici [Archive of administrative unit boundaries for statistical purposes]. Rome: ISTAT. [Accessed: 3 Oct 2018]. Italian. Available from: https://www.istat.it/it/archivio/124086

[19] World Health Organization Regional Office for Europe (WHO/Europe). Surveillance guidelines for measles, rubella and congenital rubella syndrome in the WHO European Region, update December 2012. Copenhagen: WHO/Europe; 2012. Available from: http://www.euro.who.int/en/health-topics/communicable-diseases/measles-and-rubella/publications/2012/surveillance-guidelines-for-measles,-rubella-and-congenital-rubella-syndrome-in-the-who-european-region,-update-december-2012 23762964

[20] D’Ancona F Gianfredi V Riccardo F Iannazzo S Immunisation Registries at regional level in Italy and the roadmap for a future Italian National Registry. Ann Ig. 2018;30(2):77-85. 29465145 10.7416/ai.2018.2199

[21] Eurostat RAMON - Reference And Management Of Nomenclatures. Degree of Urbanisation (DEGURBA) - Local Administrative Units. Brussels: European Commission. [Accessed: 4 Apr 2018]. Available from: http://ec.europa.eu/eurostat/ramon/miscellaneous/index.cfm?TargetUrl=DSP_DEGURBA

[22] Caranci N, De Felici P, Giuliano G, Mancini F, Rosano A. Utilizzo degli indici di deprivazione per orientare le politiche pubbliche di contrasto alla povertà. [Use of deprivation index to inform public policies against poverty]. ISFOL; 2016. Italian. Available from: http://isfoloa.isfol.it/xmlui/handle/123456789/1328

[23] Minichilli F Santoro M Bianchi F Caranci N De Santis M Pasetto R [Evaluation of the use of the socioeconomic deprivation index at area level in ecological studies on environment and health]. Epidemiol Prev. 2017;41(3-4):187-96. 28929715 10.19191/EP17.3-4.P187.052

[24] Istituto nazionale di statistica (ISTAT). Principali statistiche geografiche sui comuni. [Geographical information about the municipalities]. Rome: ISTAT. [Accessed: 20 Jul 2018]. Italian. Available from: https://www.istat.it/it/archivio/156224

[25] ggplot2: An implementation of the Grammar of Graphics in R. tidyverse; 2018. Available from: https://github.com/tidyverse/ggplot2

[26] Istituto nazionale di statistica (ISTAT). Statistiche demografiche [Demographical statistics]. Rome: ISTAT. [Accessed: 15 Jan 2018]. Available from: http://demo.istat.it/pop2017/index.html

[27] van Buuren S, Groothuis-Oudshoorn K. mice: Multivariate Imputation by Chained Equations in R. J Statistical Software; 2012. Available from: https://www.jstatsoft.org/article/view/v045i03

[28] Fitzpatrick G Ward M Ennis O Johnson H Cotter S Carr MJ Use of a geographic information system to map cases of measles in real-time during an outbreak in Dublin, Ireland, 2011. Euro Surveill. 2012;17(49):20330. 10.2807/ese.17.49.20330-en 23231894

[29] Yang W Wen L Li S-L Chen K Zhang W-Y Shaman J Geospatial characteristics of measles transmission in China during 2005-2014. PLOS Comput Biol. 2017;13(4):e1005474. 10.1371/journal.pcbi.1005474 28376097 PMC5395235

[30] Salmaso S Gabutti G Rota MC Giordano C Penna C Mandolini D Pattern of susceptibility to measles in Italy. Bull World Health Organ. 2000;78(8):950-5. 10994277 PMC2560816

[31] R Core Team. R: A Language and Environment for Statistical Computing. Vienna: R Foundation for Statistical Computing; 2019. Available from: https://www.R-project.org

[32] RStudio Team. RStudio: Integrated Development Environment for R. Boston: RStudio Inc.; 2019. Available from: http://www.rstudio.com/

[33] Ciofi Degli Atti ML Salmaso S Bella A Arigliani R Gangemi M Chiamenti G Pediatric sentinel surveillance of vaccine-preventable diseases in Italy. Pediatr Infect Dis J. 2002;21(8):763-8. 10.1097/00006454-200208000-00013 12192166

[34] Ciofi degli Atti ML Rota MC Mandolini D Bella A Gabutti G Crovari P Assessment of varicella underreporting in Italy. Epidemiol Infect. 2002;128(3):479-84. 10.1017/S0950268802006878 12113493 PMC2869845

[35] Filia A Bella A Del Manso M Baggieri M Magurano F Rota MC Ongoing outbreak with well over 4,000 measles cases in Italy from January to end August 2017 - what is making elimination so difficult? Euro Surveill. 2017;22(37):30614. 10.2807/1560-7917.ES.2017.22.37.30614 28933342 PMC5607657

[36] Perry RT Halsey NA The clinical significance of measles: a review. J Infect Dis. 2004;189(s1) Suppl 1;S4-16. 10.1086/377712 15106083

[37] Parent du Châtelet I Antona D Freymuth F Muscat M Halftermeyer-Zhou F Maine C Spotlight on measles 2010: update on the ongoing measles outbreak in France, 2008-2010. Euro Surveill. 2010;15(36):19656. 20843472

[38] Rivadeneira MF Bassanesi SL Fuchs SC Role of health determinants in a measles outbreak in Ecuador: a case-control study with aggregated data. BMC Public Health. 2018;18(1):269. 10.1186/s12889-018-5163-9 29458349 PMC5819223

[39] Chong KC Zhang C Jia KM Zee BCY Luo T Wang L Targeting adults for supplementary immunization activities of measles control in central China: A Mathematical Modelling Study. Sci Rep. 2018;8(1):16124. 10.1038/s41598-018-34461-0 30382120 PMC6208397

[40] Keenan A Ghebrehewet S Vivancos R Seddon D MacPherson P Hungerford D Measles outbreaks in the UK, is it when and where, rather than if? A database cohort study of childhood population susceptibility in Liverpool, UK. BMJ Open. 2017;7(3):e014106. 10.1136/bmjopen-2016-014106 28363926 PMC5387959

[41] Hoes J Boef AGC Knol MJ de Melker HE Mollema L van der Klis FRM Socioeconomic status is associated with antibody levels against vaccine preventable diseases in the Netherlands. Front Public Health. 2018;6:209. 10.3389/fpubh.2018.00209 30140666 PMC6094970

[42] Fielding JE Bolam B Danchin MH Immunisation coverage and socioeconomic status - questioning inequity in the ‘No Jab, No Pay’ policy. Aust N Z J Public Health. 2017;41(5):455-7. 10.1111/1753-6405.12676 28664595

[43] Giambi C Fabiani M D’Ancona F Ferrara L Fiacchini D Gallo T Parental vaccine hesitancy in Italy - Results from a national survey. Vaccine. 2018;36(6):779-87. 10.1016/j.vaccine.2017.12.074 29325822

[44] Burioni R Odone A Signorelli C Lessons from Italy’s policy shift on immunization. Nature. 2018;555(7694):30. 10.1038/d41586-018-02267-9 29493608

