# Supplementary material for: Spatiotemporal distribution and determinants of measles incidence during a large outbreak, Italy, September 2016 to July 2018
Source: Euro Surveill. 2019 Apr 25;24(17):1800679. doi: 10.2807/1560-7917.ES.2019.24.17.1800679 (PMC6628759; doi:10.2807/1560-7917.ES.2019.24.17.1800679)
Supplement: SupplementaryMaterial_Figures-and-Tables [file 18-00679_FILIA_SupplementaryMaterial_Figures-and-Tables.pdf]

Supplementary material

This supplementary material is hosted by *Eurosurveillance* as supporting information alongside the article “Spatiotemporal distribution and determinants of measles incidence during a large outbreak, Italy, September 2016 to July 2018” on behalf of the authors who remain responsible for the accuracy and appropriateness of the content. The same standards for ethics, copyright, attributions and permissions as for the article apply. *Eurosurveillance* is not responsible for the maintenance of any links or email addresses provided therein.

Note: The data included in this analysis were retrieved on September 6, 2018.

Figure S 1 Map of the regions/autonomous provinces of Italy.

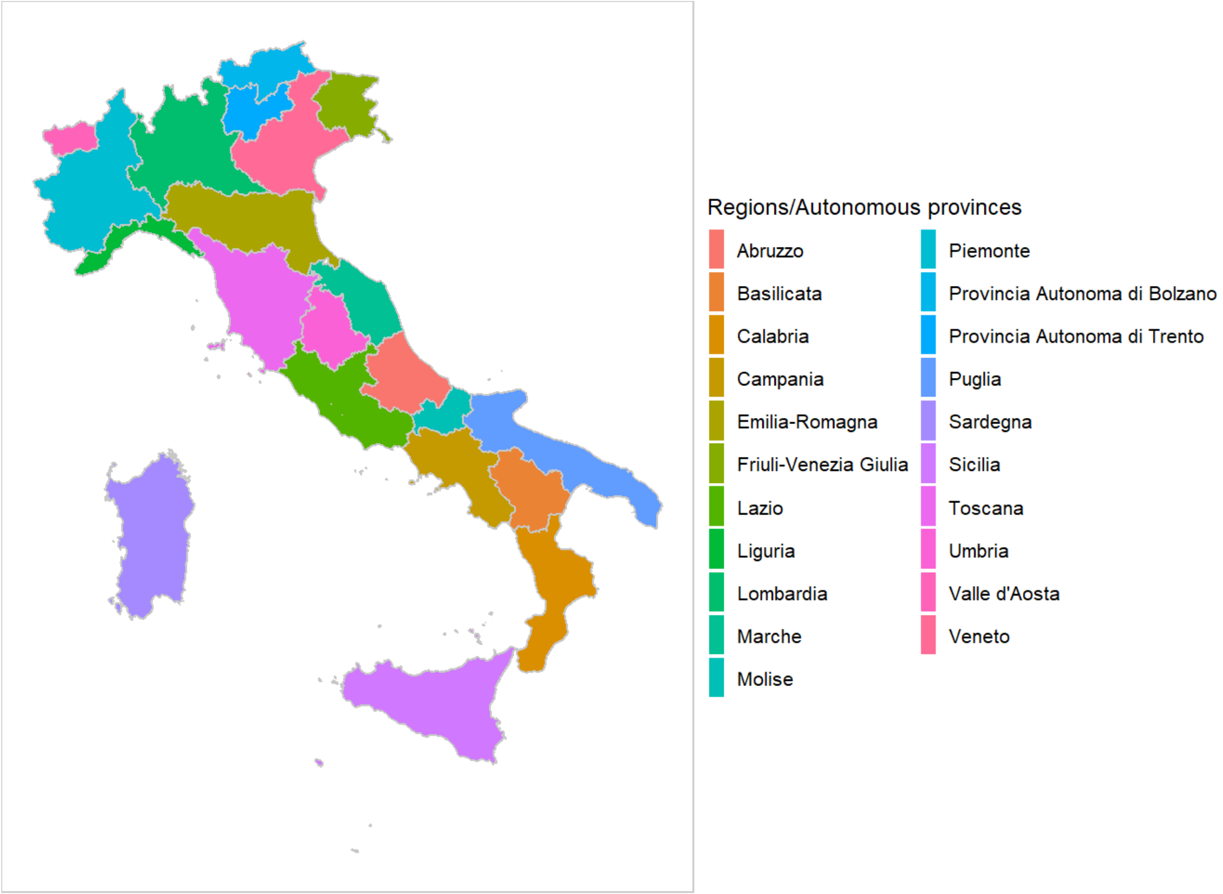

Figure S 2 Flow chart of the data included in the analysis and a summary of the available information of the place of onset.

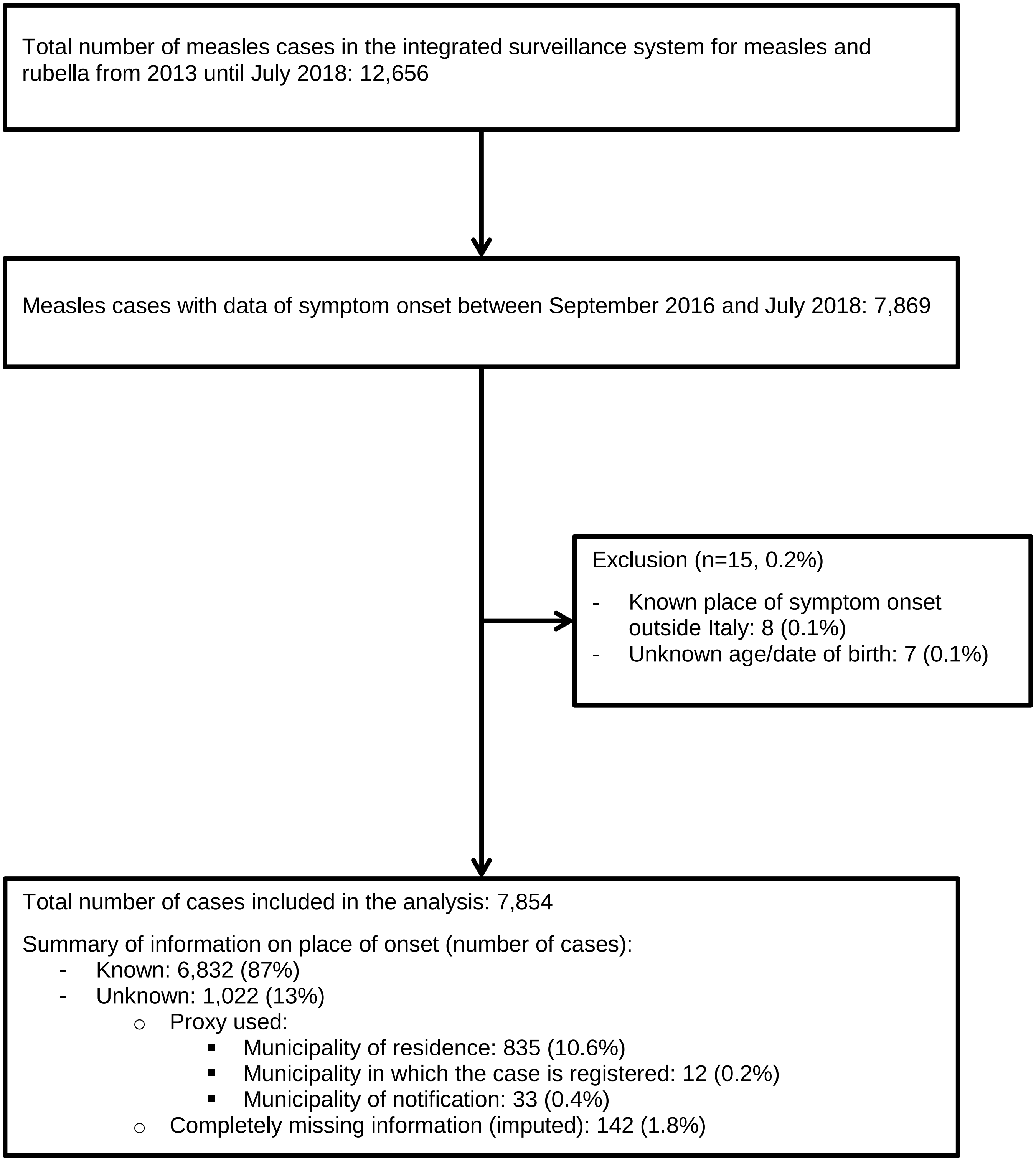

Figure S 3 Regional measles incidence (per 1,000,000 per year) of measles between September 2016 and July 2018 for the complete study population (0-50 years-old), for infants (<1 year-old), and by age group.

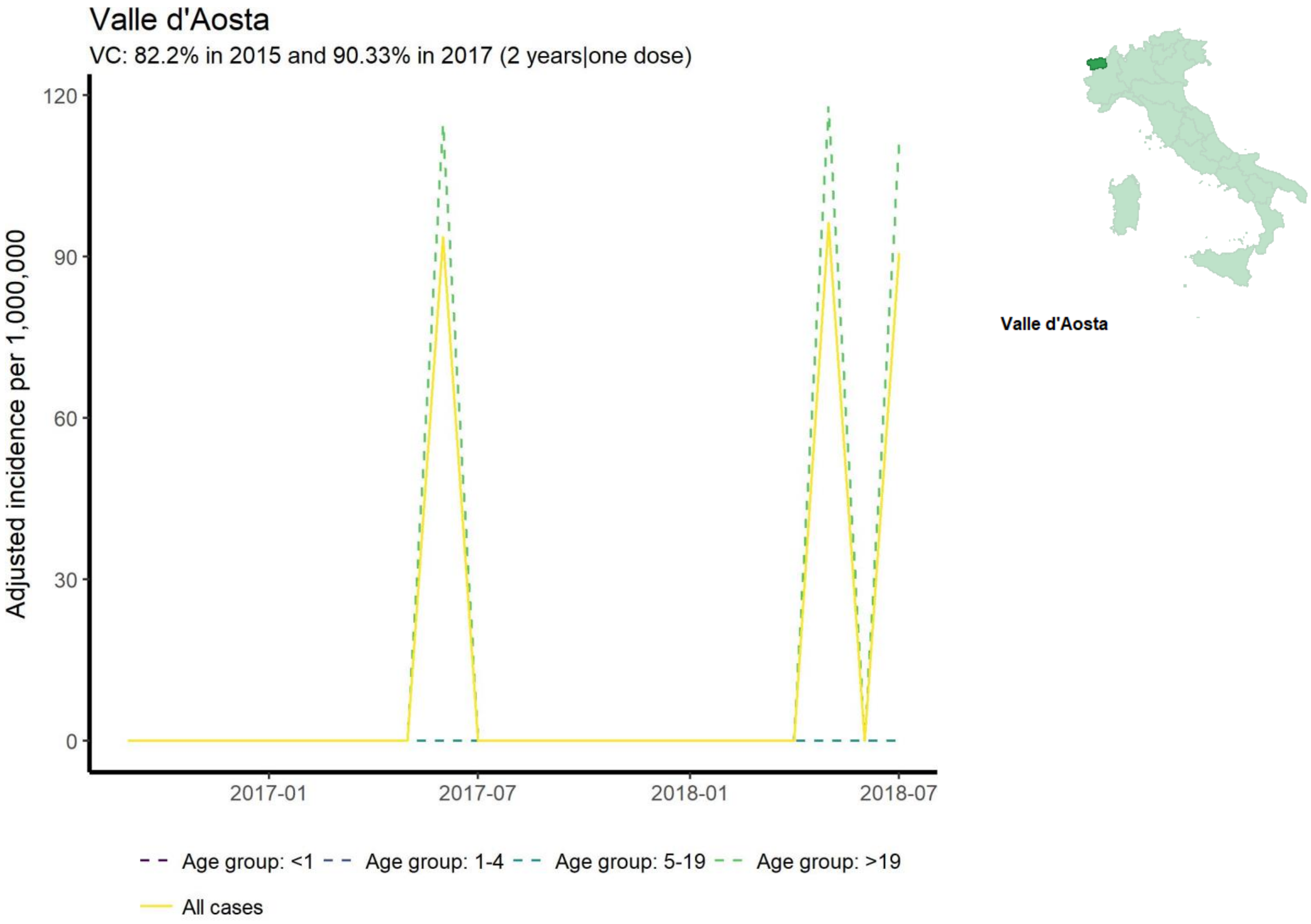

Piemonte

VC: 88.7% in 2015 and 94.72% in 2017 (2 years|one dose)

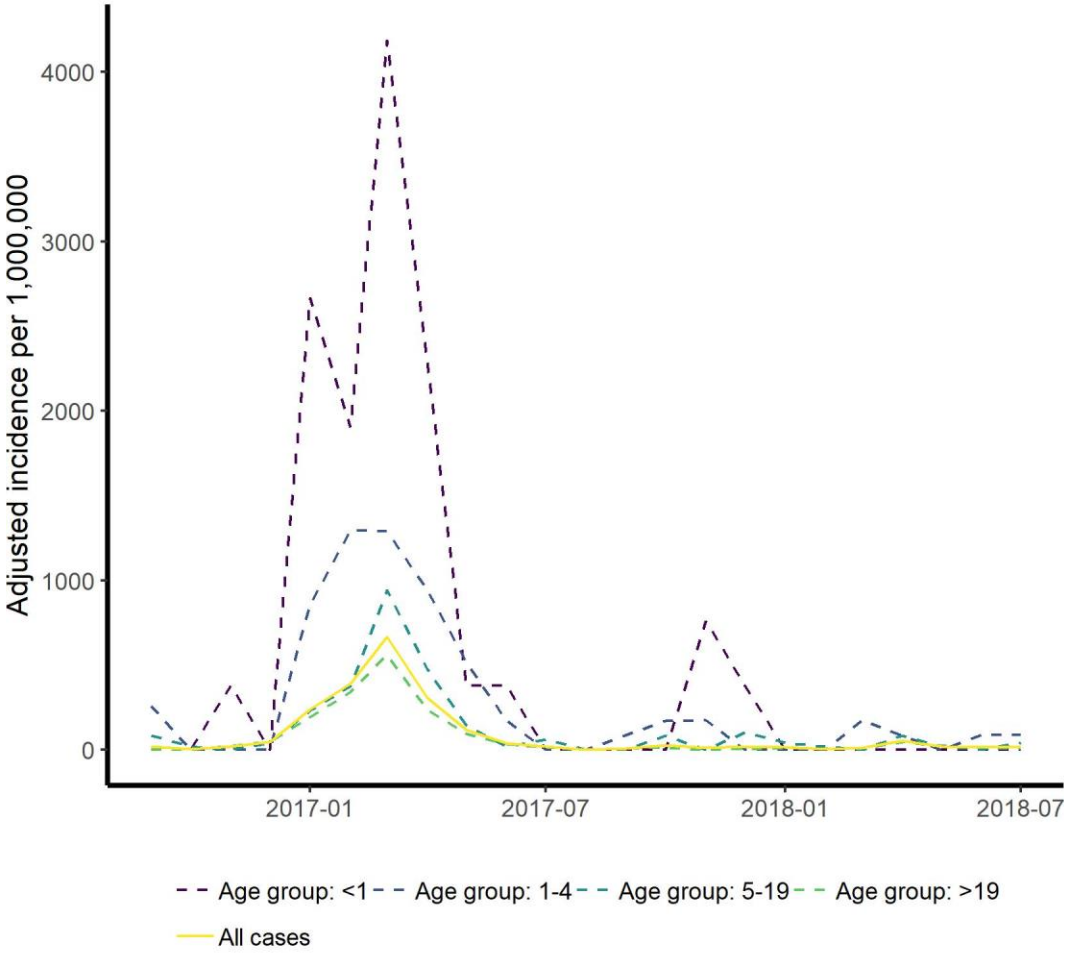

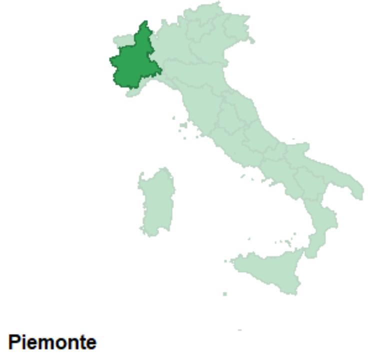

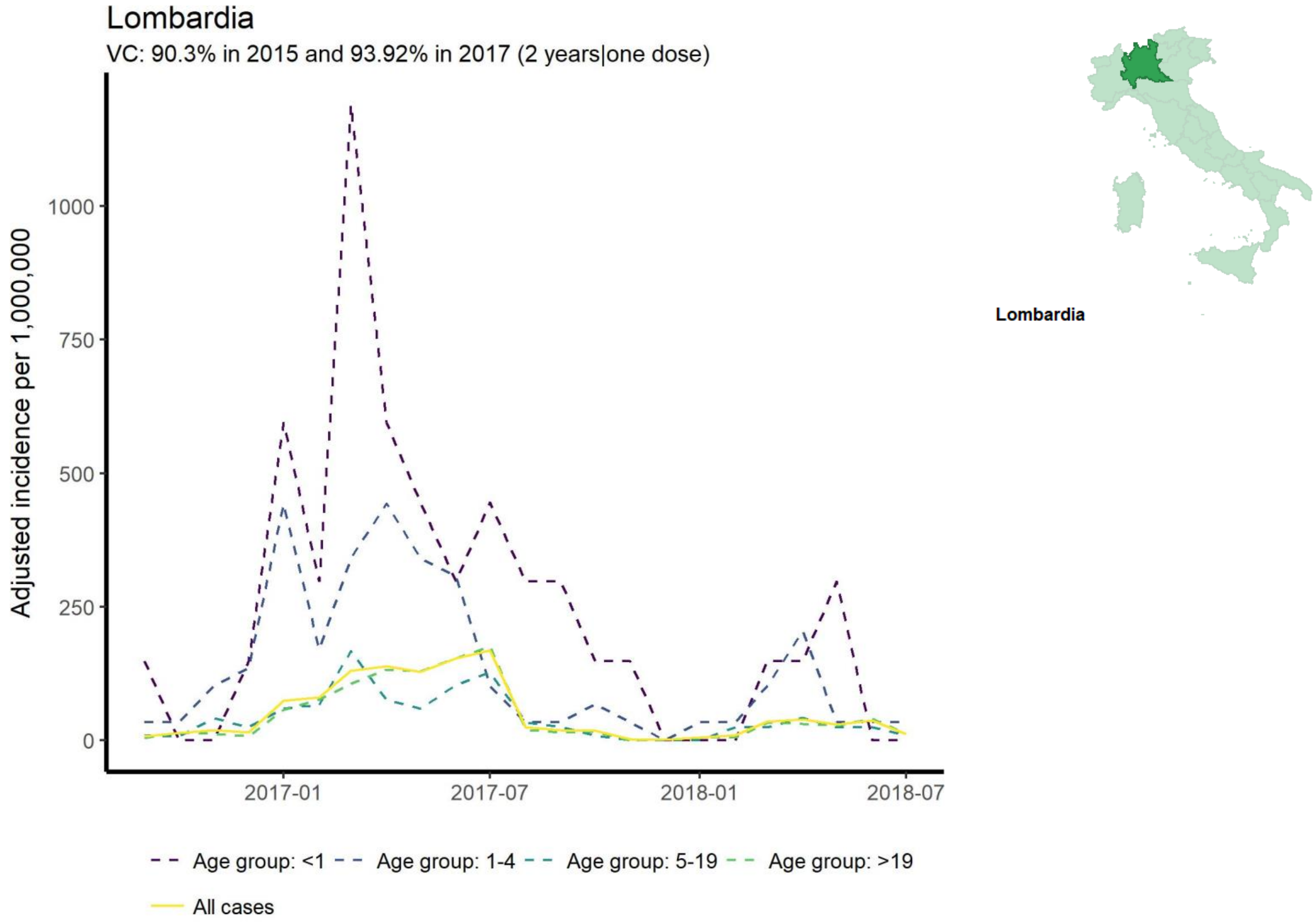
Lombardia
VC: 90.3% in 2015 and 93.92% in 2017 (2 years|one dose)
Adjusted incidence per 1,000,000
1000
750
500
250
0
2017-01
2017-07
2018-01
2018-07
Age group: <1
Age group: 1-4
Age group: 5-19
Age group: >19
All cases
Lombardia

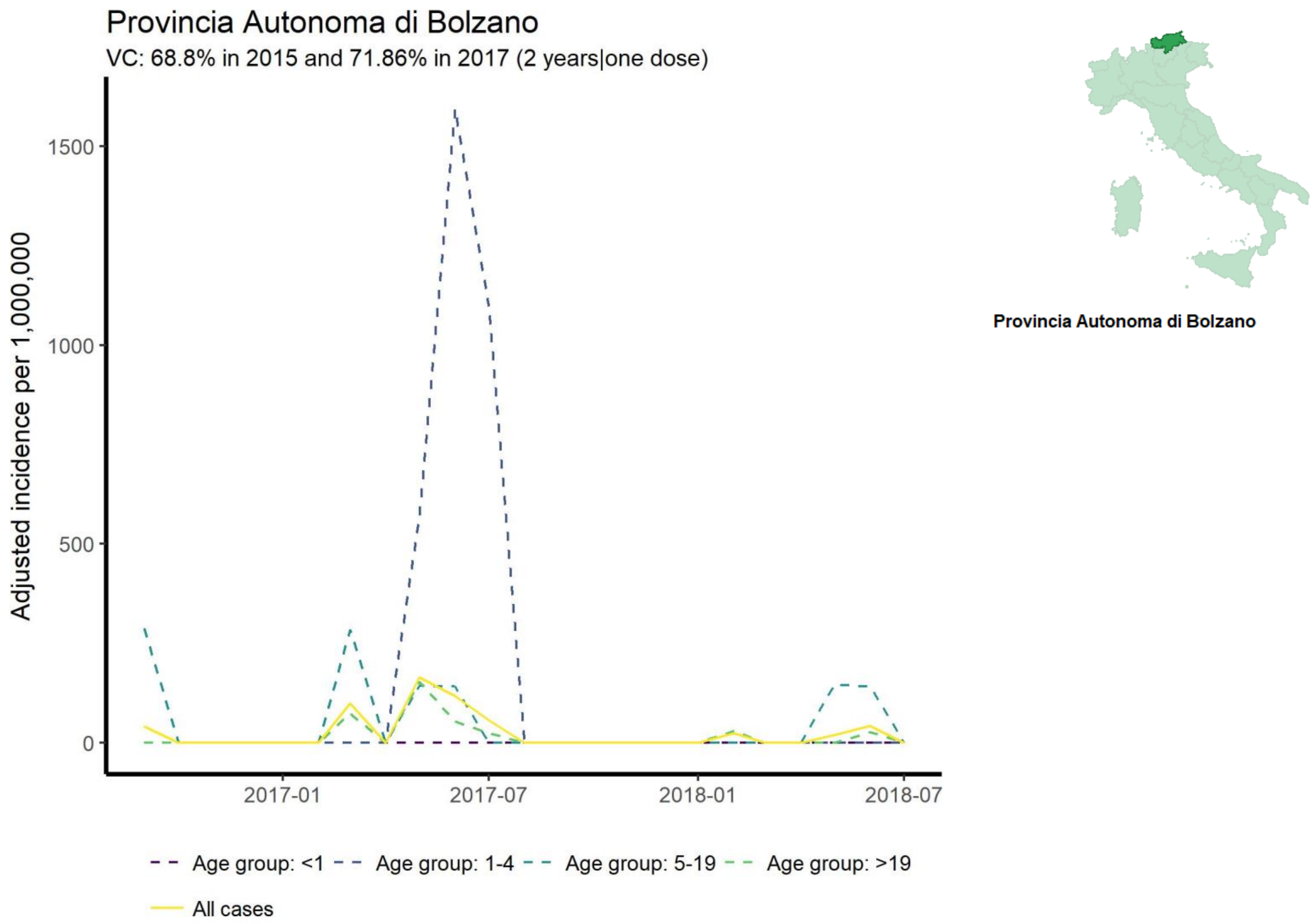
Provincia Autonoma di Bolzano
VC: 68.8% in 2015 and 71.86% in 2017 (2 years|one dose)
Adjusted incidence per 1,000,000
1500
1000
500
0
2017-01
2017-07
2018-01
2018-07
Age group: <1
Age group: 1-4
Age group: 5-19
Age group: >19
All cases
Provincia Autonoma di Bolzano

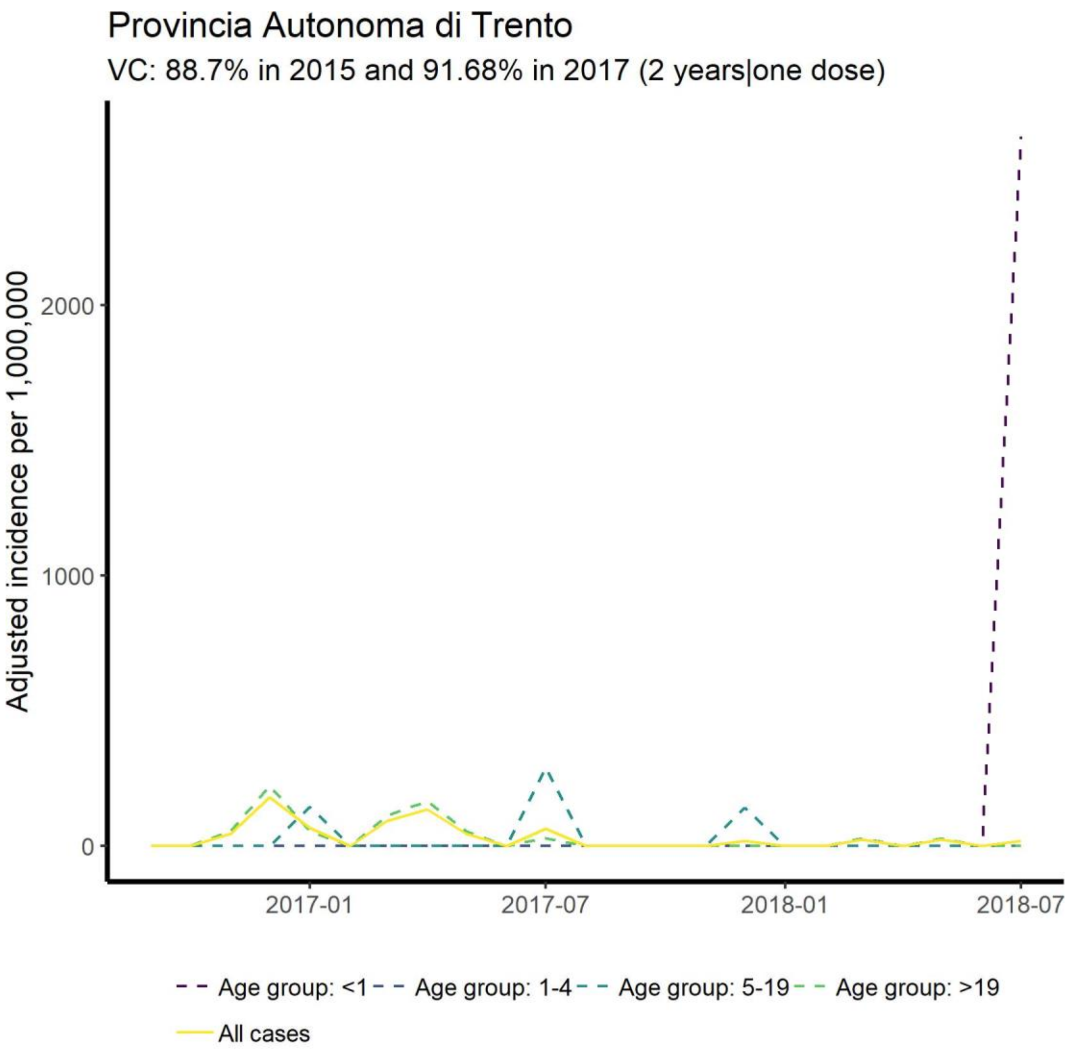

Provincia Autonoma di Trento
VC: 88.7% in 2015 and 91.68% in 2017 (2 years|one dose)
Adjusted incidence per 1,000,000
2000
1000
0
2017-01
2017-07
2018-01
2018-07
Age group: <1
Age group: 1-4
Age group: 5-19
Age group: >19
All cases

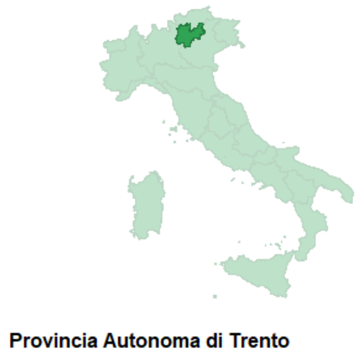

Provincia Autonoma di Trento

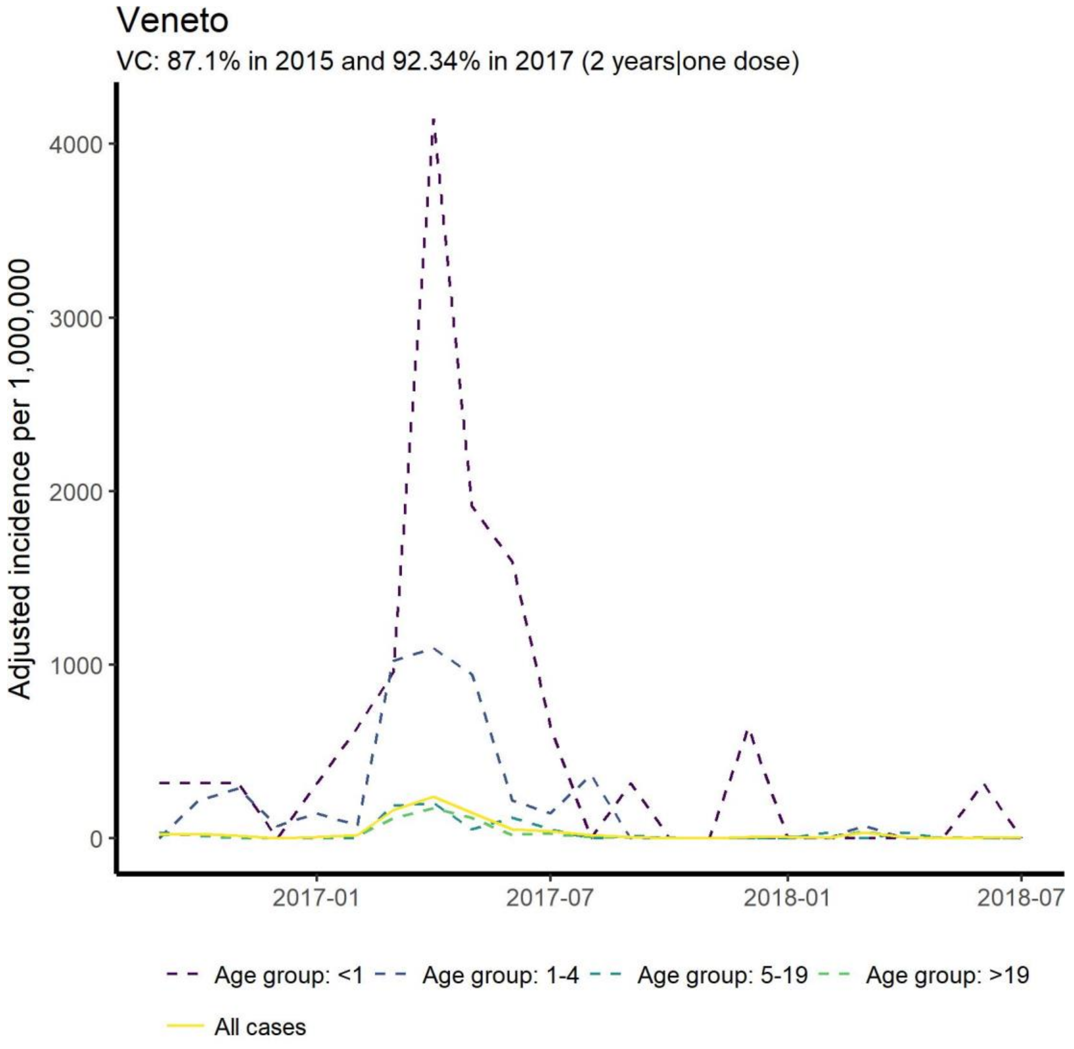
Veneto
VC: 87.1% in 2015 and 92.34% in 2017 (2 years|one dose)
Adjusted incidence per 1,000,000
4000
3000
2000
1000
0
2017-01
2017-07
2018-01
2018-07
Age group: <1
Age group: 1-4
Age group: 5-19
Age group: >19
All cases

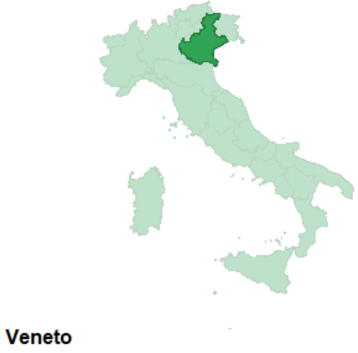
Veneto

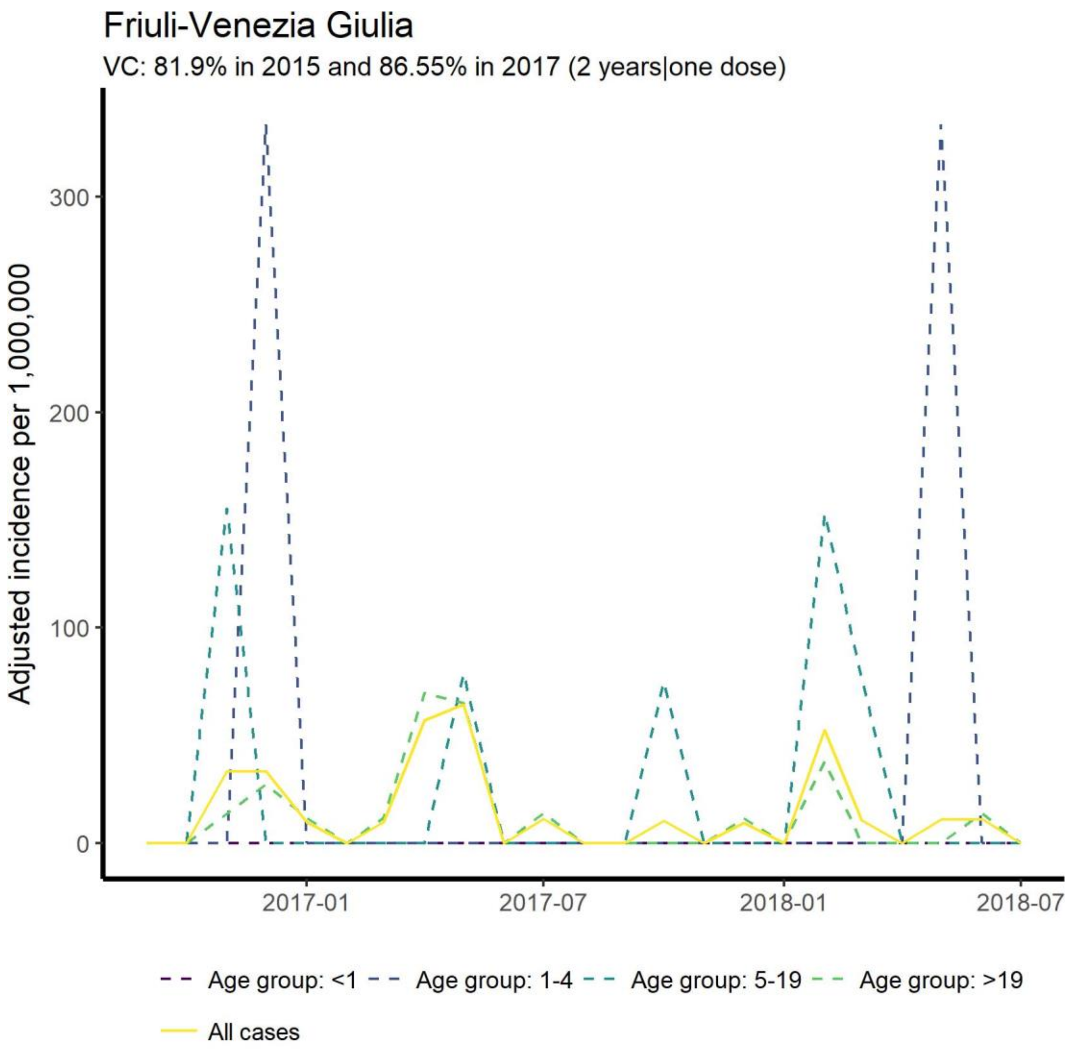
Friuli-Venezia Giulia
VC: 81.9% in 2015 and 86.55% in 2017 (2 years|one dose)
Adjusted incidence per 1,000,000
300
200
100
0
2017-01
2017-07
2018-01
2018-07
Age group: <1
Age group: 1-4
Age group: 5-19
Age group: >19
All cases

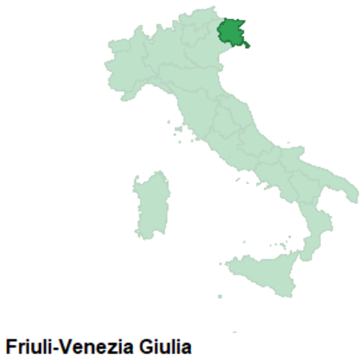
Friuli-Venezia Giulia

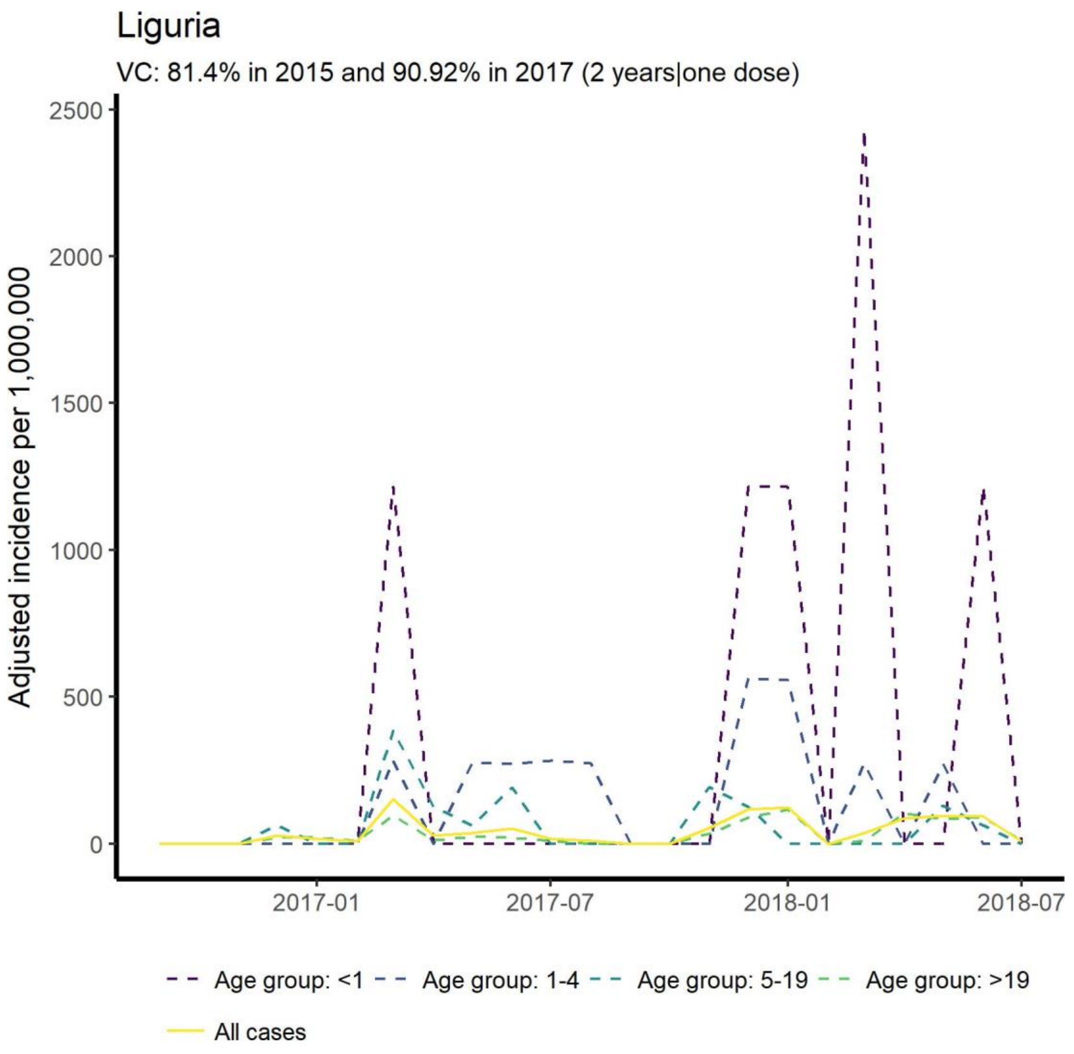

Liguria
VC: 81.4% in 2015 and 90.92% in 2017 (2 years|one dose)
Adjusted incidence per 1,000,000
2500
2000
1500
1000
500
0
2017-01
2017-07
2018-01
2018-07
Age group: <1
Age group: 1-4
Age group: 5-19
Age group: >19
All cases

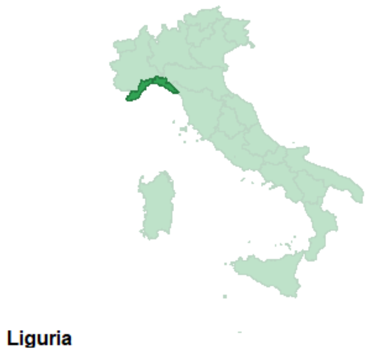

Liguria

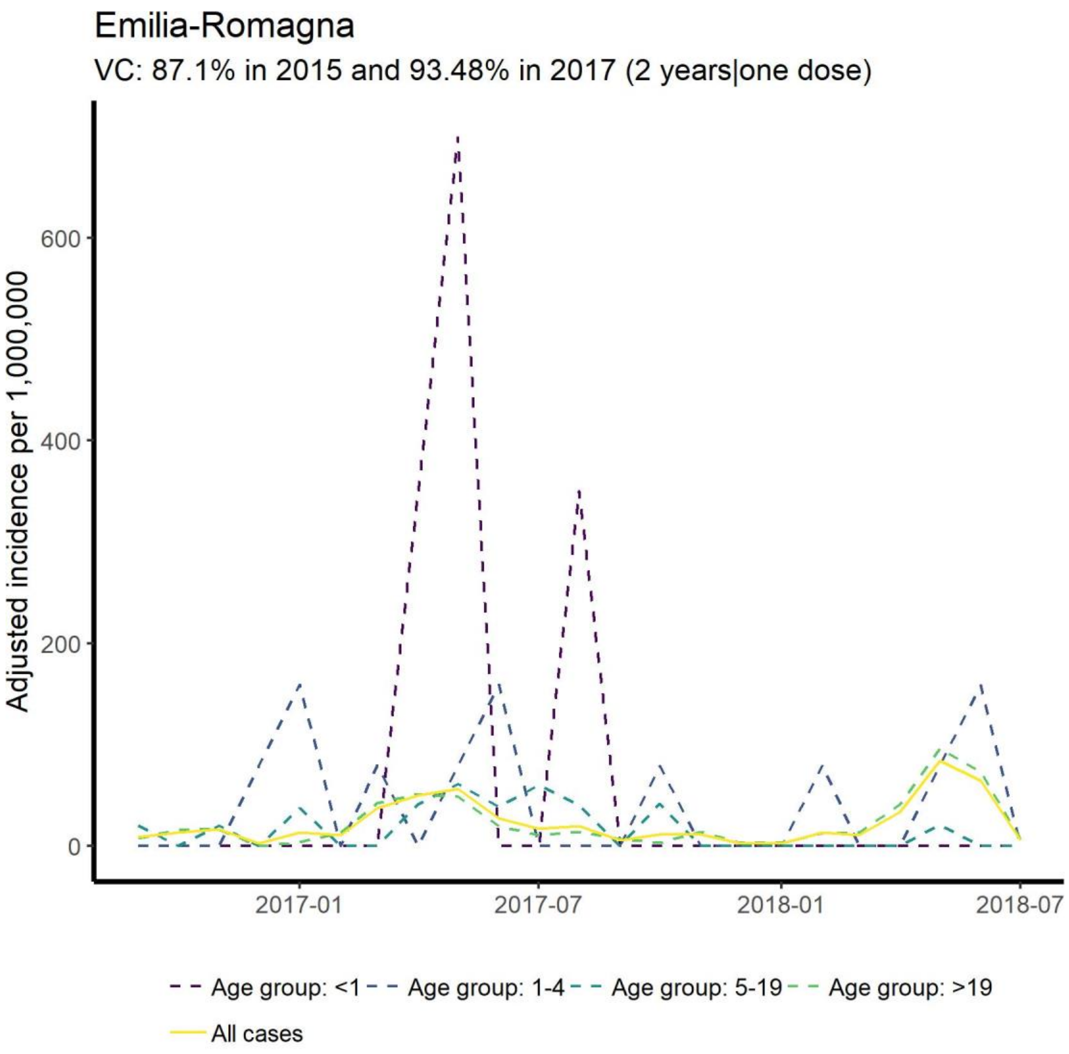

Emilia-Romagna
VC: 87.1% in 2015 and 93.48% in 2017 (2 years|one dose)
Adjusted incidence per 1,000,000
600
400
200
0
2017-01
2017-07
2018-01
2018-07
Age group: <1
Age group: 1-4
Age group: 5-19
Age group: >19
All cases

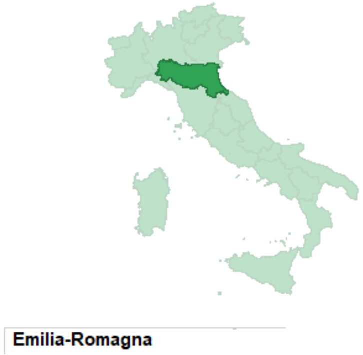

Emilia-Romagna

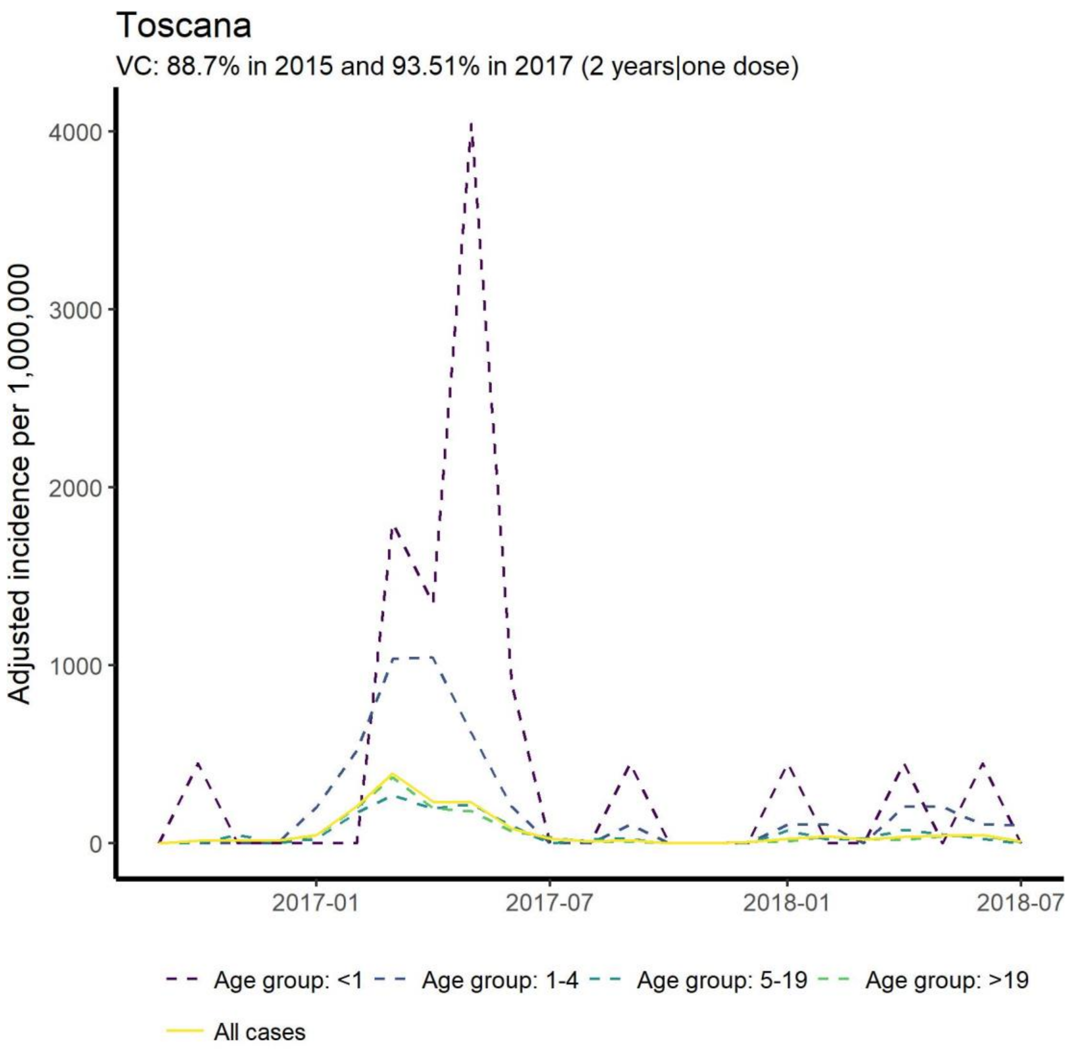
Toscana
VC: 88.7% in 2015 and 93.51% in 2017 (2 years|one dose)
Adjusted incidence per 1,000,000
4000
3000
2000
1000
0
2017-01
2017-07
2018-01
2018-07
Age group: <1
Age group: 1-4
Age group: 5-19
Age group: >19
All cases

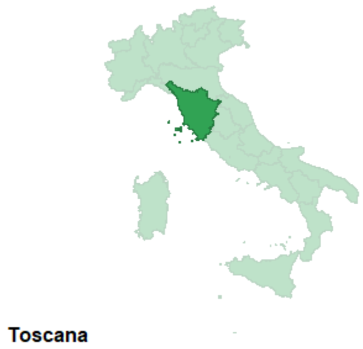
Toscana

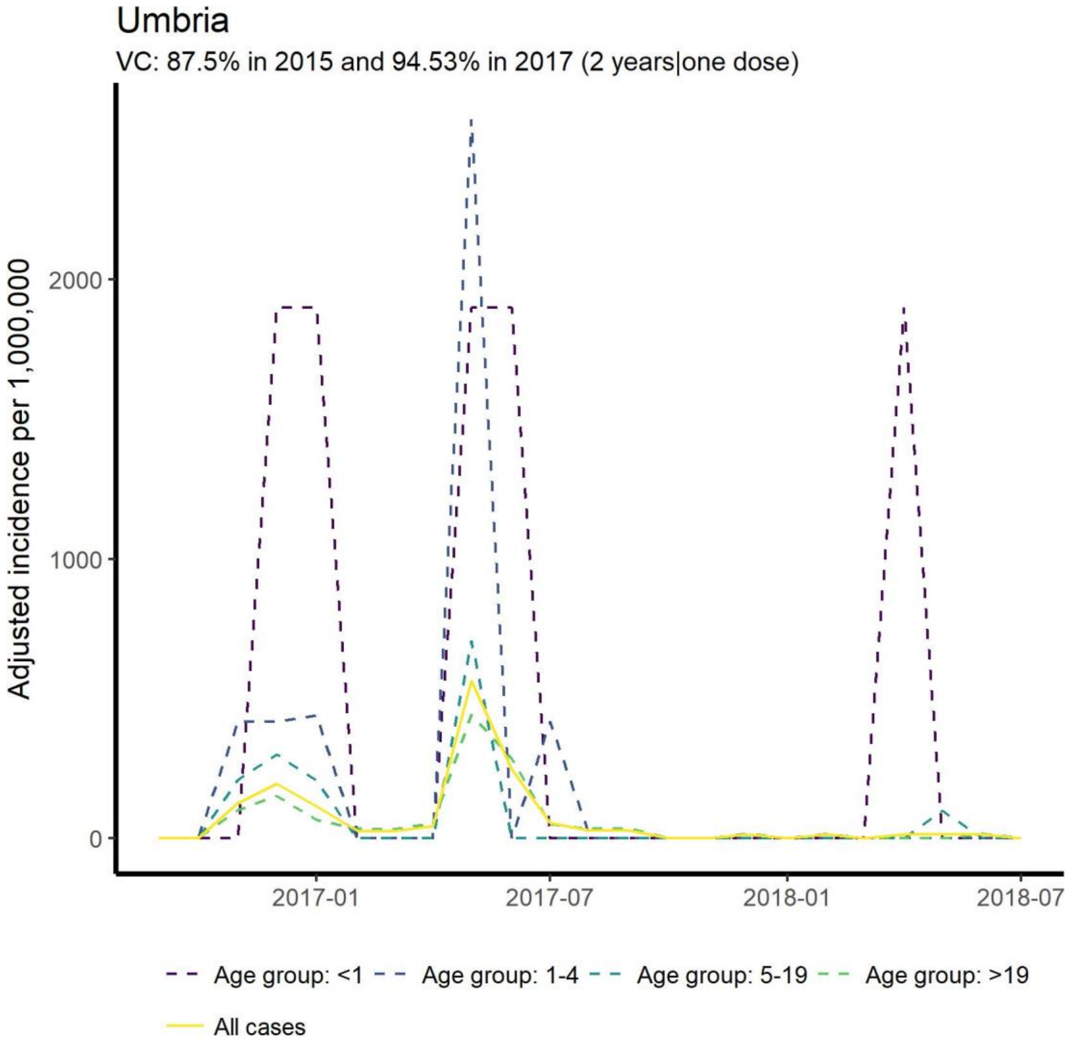
Umbria
VC: 87.5% in 2015 and 94.53% in 2017 (2 years|one dose)
Adjusted incidence per 1,000,000
2000
1000
0
2017-01
2017-07
2018-01
2018-07
Age group: <1
Age group: 1-4
Age group: 5-19
Age group: >19
All cases

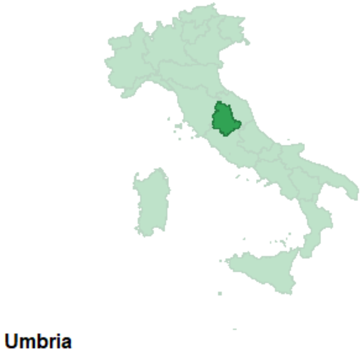
Umbria

Marche

VC: 79.9% in 2015 and 88.21% in 2017 (2 years|one dose)

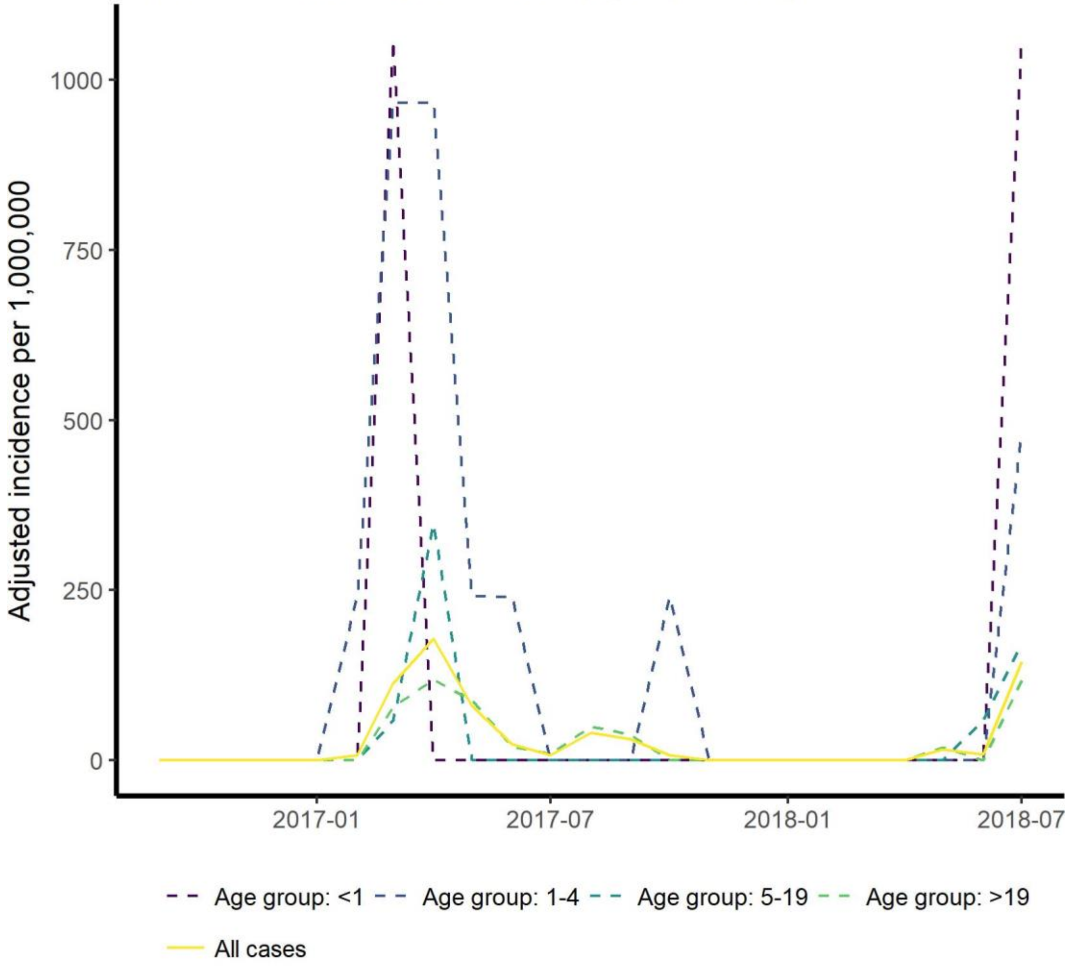

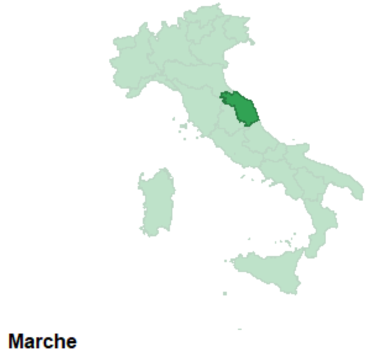

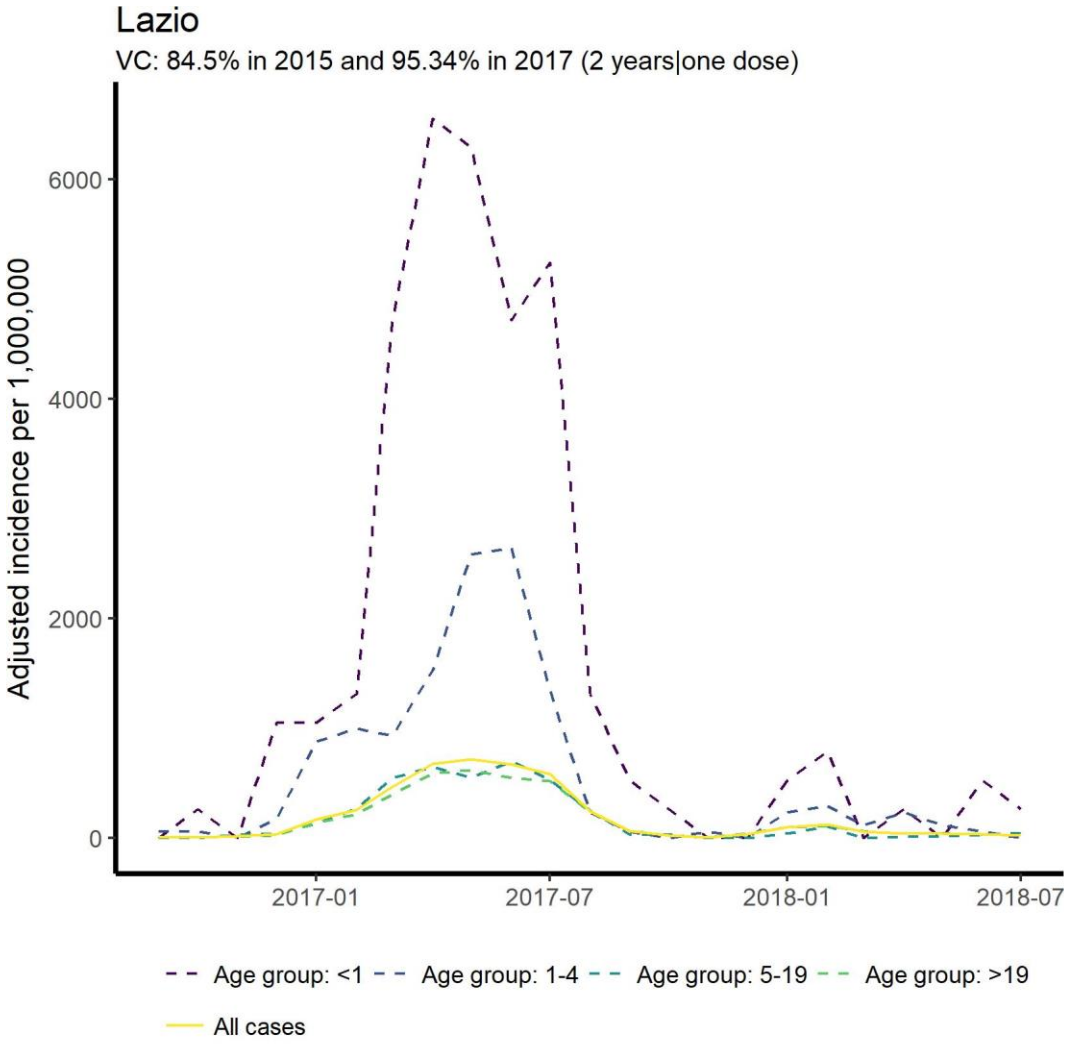
Lazio
VC: 84.5% in 2015 and 95.34% in 2017 (2 years|one dose)
Adjusted incidence per 1,000,000
6000
4000
2000
0
2017-01
2017-07
2018-01
2018-07
Age group: <1
Age group: 1-4
Age group: 5-19
Age group: >19
All cases

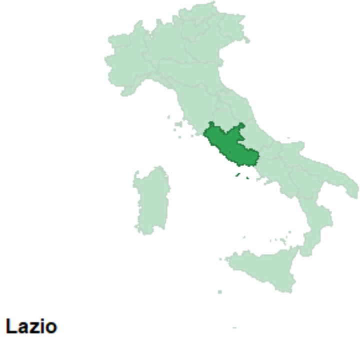
Lazio

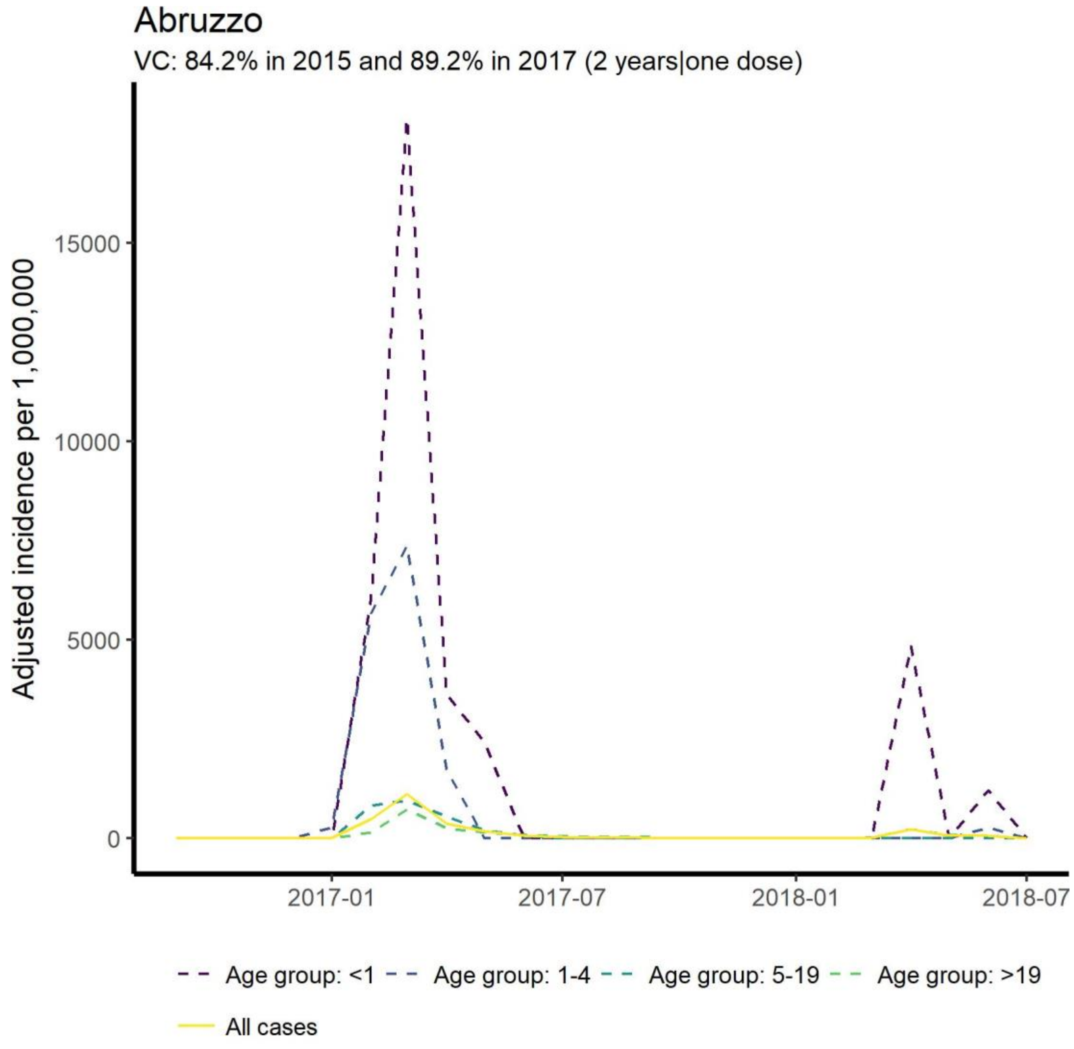
Abruzzo
VC: 84.2% in 2015 and 89.2% in 2017 (2 years|one dose)
Adjusted incidence per 1,000,000
15000
10000
5000
0
2017-01
2017-07
2018-01
2018-07
Age group: <1
Age group: 1-4
Age group: 5-19
Age group: >19
All cases

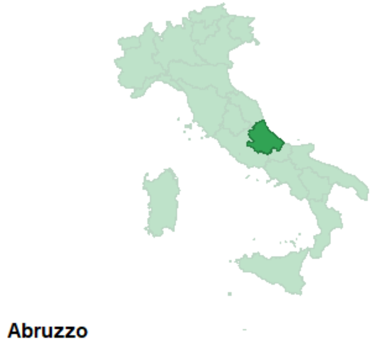
Abruzzo

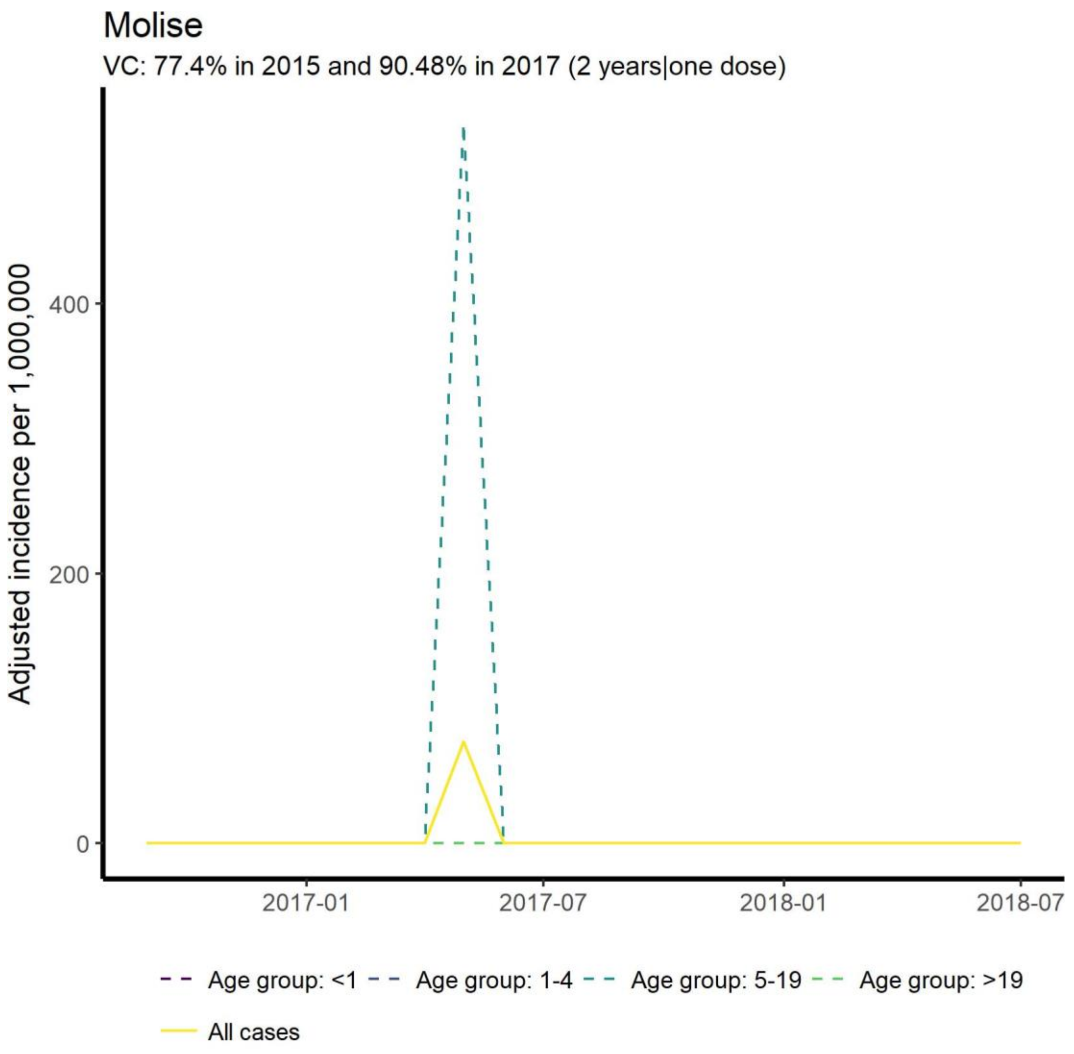
Molise
VC: 77.4% in 2015 and 90.48% in 2017 (2 years|one dose)
Adjusted incidence per 1,000,000
400
200
0
2017-01
2017-07
2018-01
2018-07
Age group: <1
Age group: 1-4
Age group: 5-19
Age group: >19
All cases

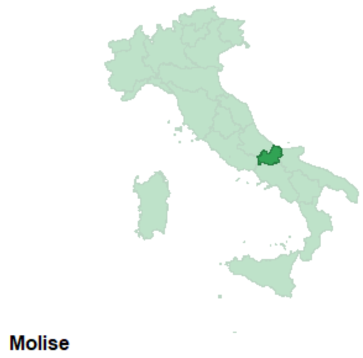
Molise

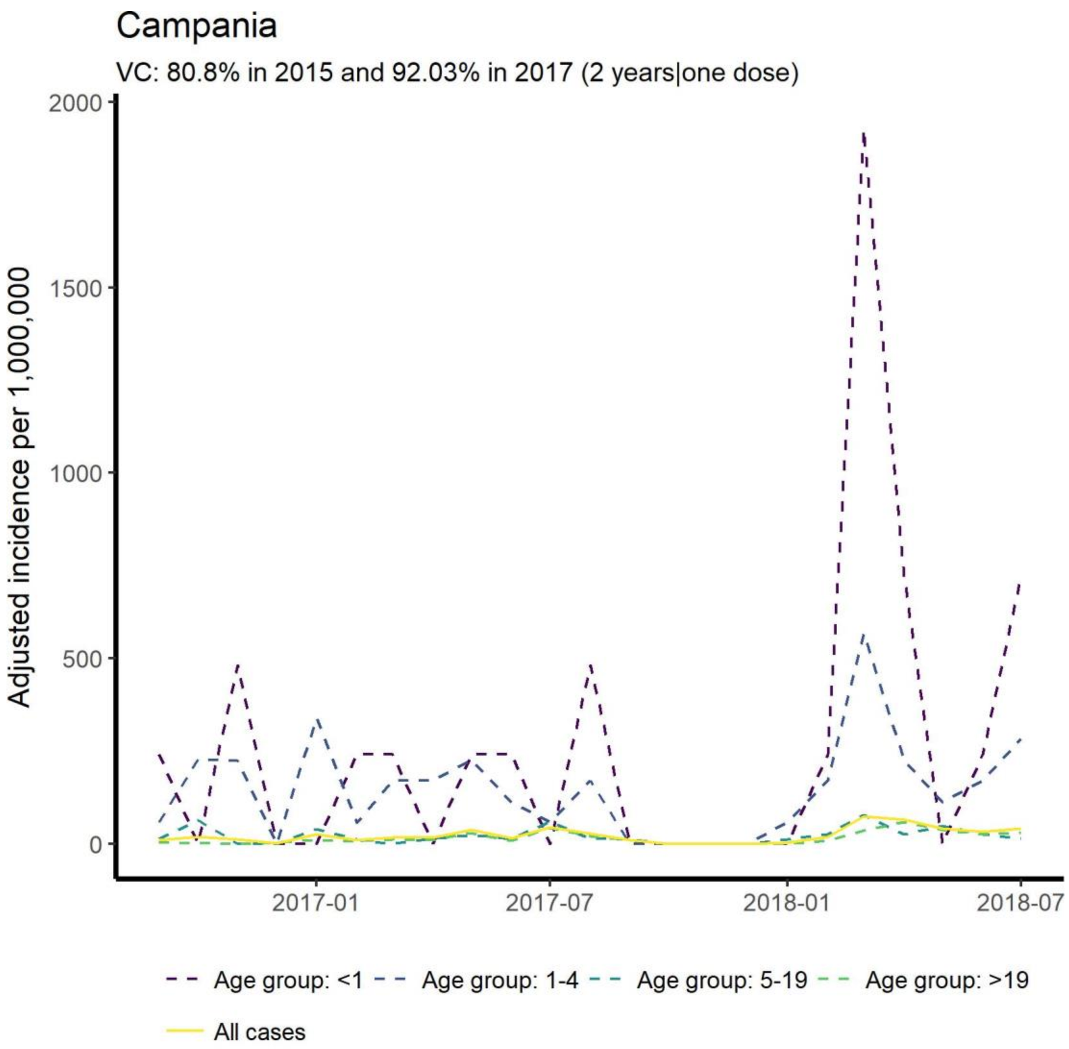
Campania
VC: 80.8% in 2015 and 92.03% in 2017 (2 years|one dose)
Adjusted incidence per 1,000,000
2000
1500
1000
500
0
2017-01
2017-07
2018-01
2018-07
Age group: <1
Age group: 1-4
Age group: 5-19
Age group: >19
All cases

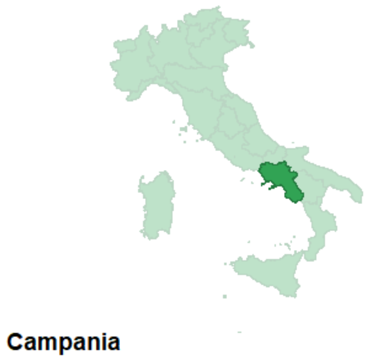
Campania

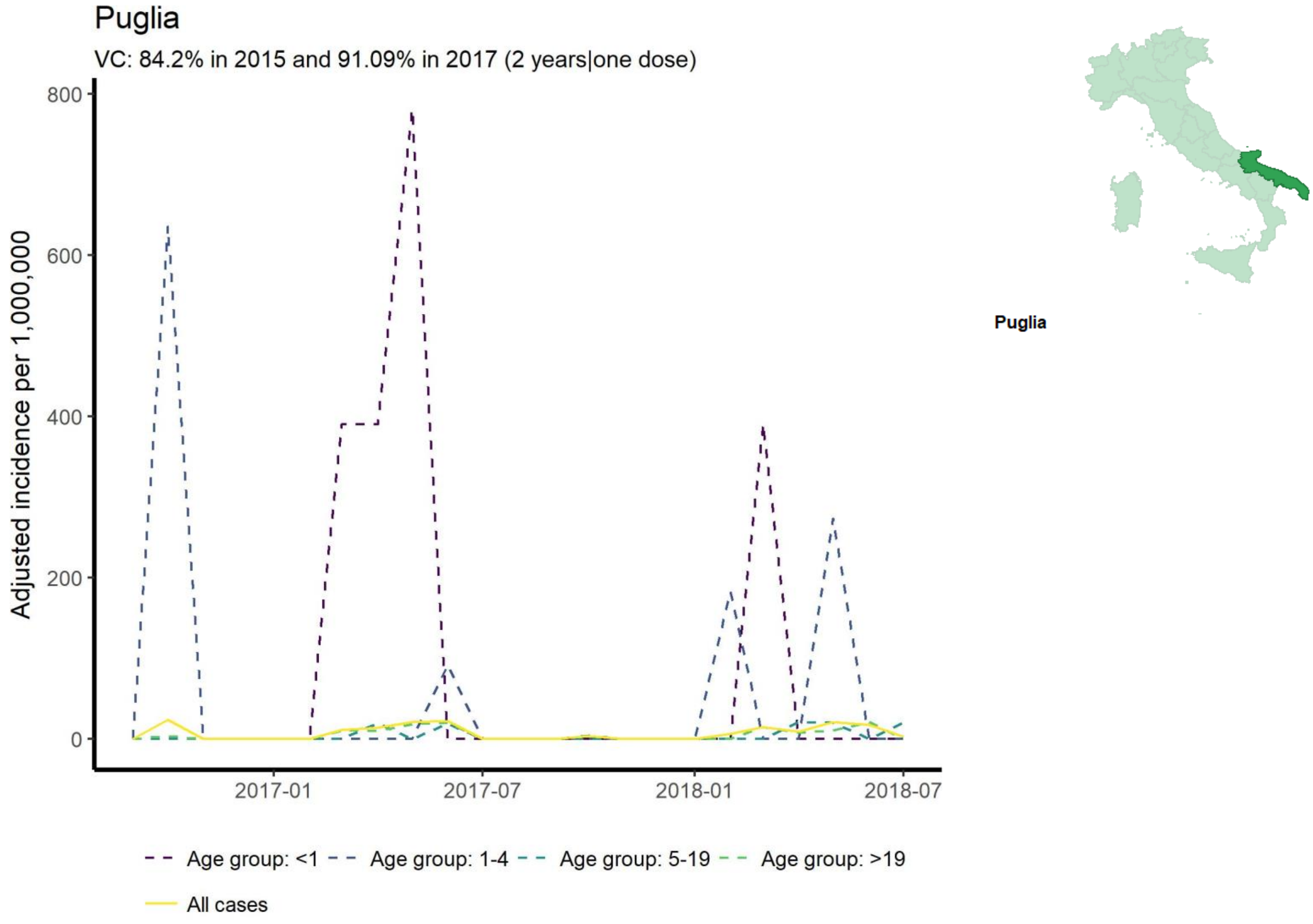
Puglia
VC: 84.2% in 2015 and 91.09% in 2017 (2 years|one dose)
Adjusted incidence per 1,000,000
800
600
400
200
0
2017-01
2017-07
2018-01
2018-07
Age group: <1
Age group: 1-4
Age group: 5-19
Age group: >19
All cases
Puglia

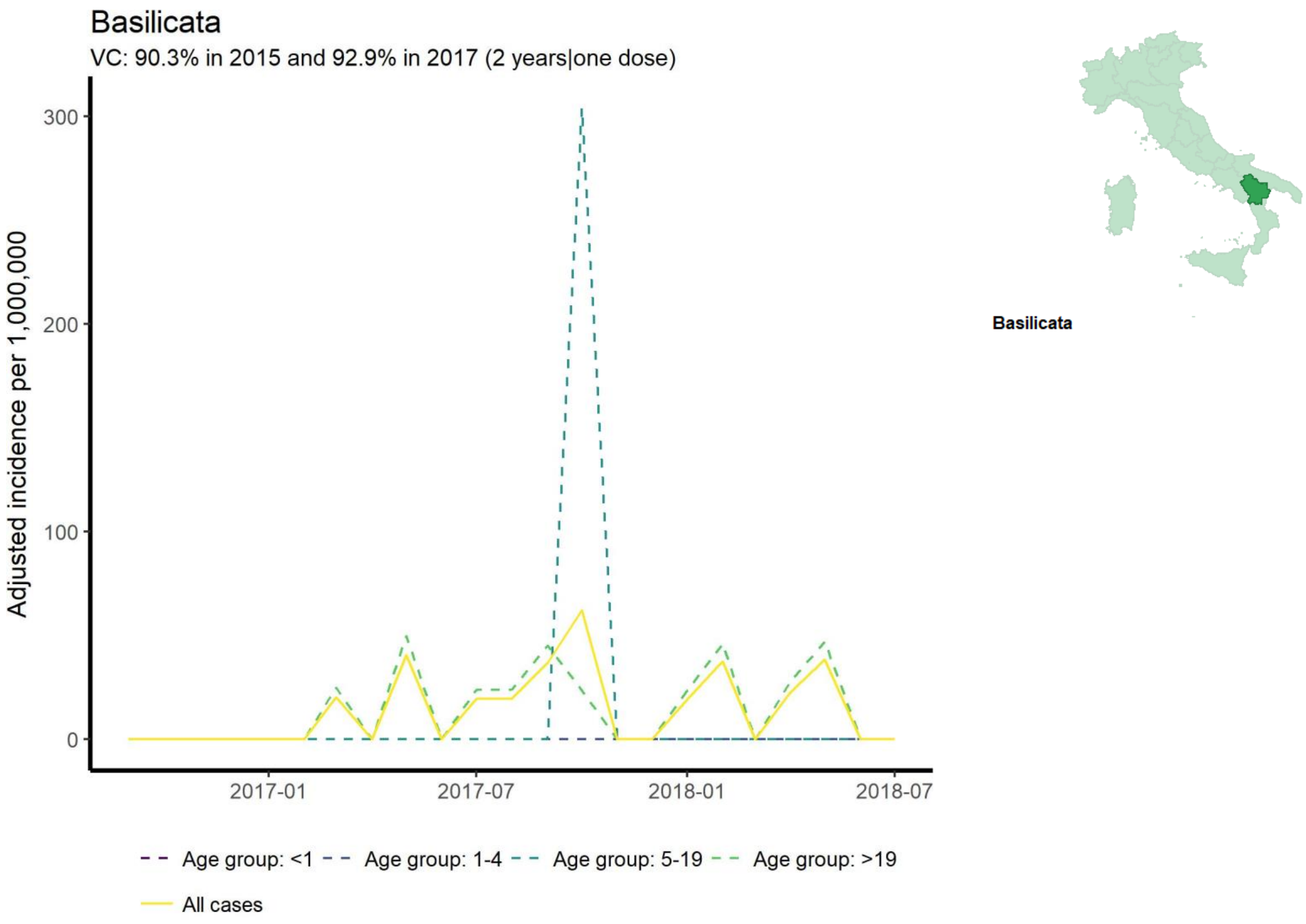
Basilicata
VC: 90.3% in 2015 and 92.9% in 2017 (2 years|one dose)
Adjusted incidence per 1,000,000
300
200
100
0
2017-01
2017-07
2018-01
2018-07
Age group: <1
Age group: 1-4
Age group: 5-19
Age group: >19
All cases
Basilicata

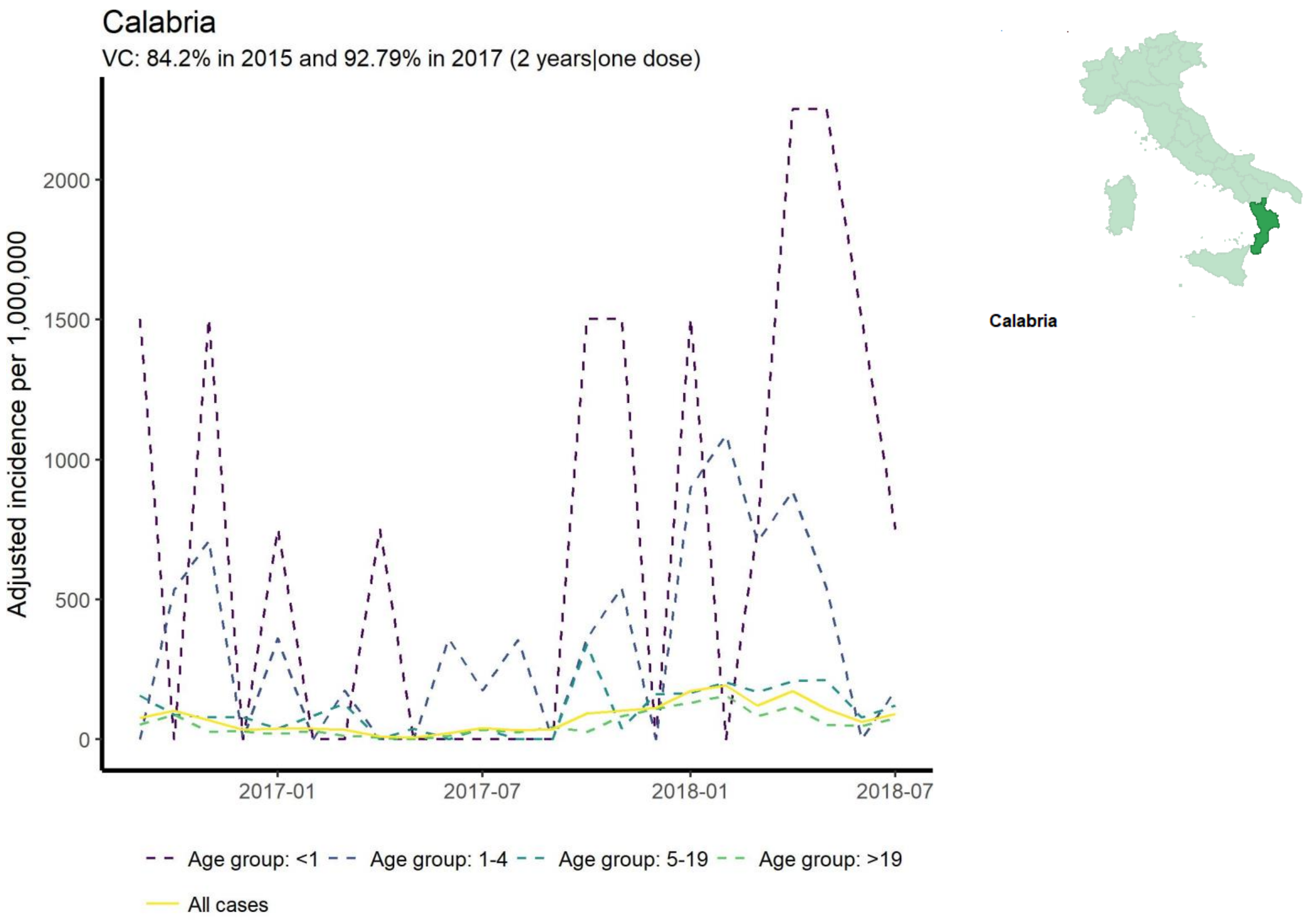
Calabria
VC: 84.2% in 2015 and 92.79% in 2017 (2 years|one dose)
Adjusted incidence per 1,000,000
2000
1500
1000
500
0
2017-01
2017-07
2018-01
2018-07
Age group: <1
Age group: 1-4
Age group: 5-19
Age group: >19
All cases
Calabria

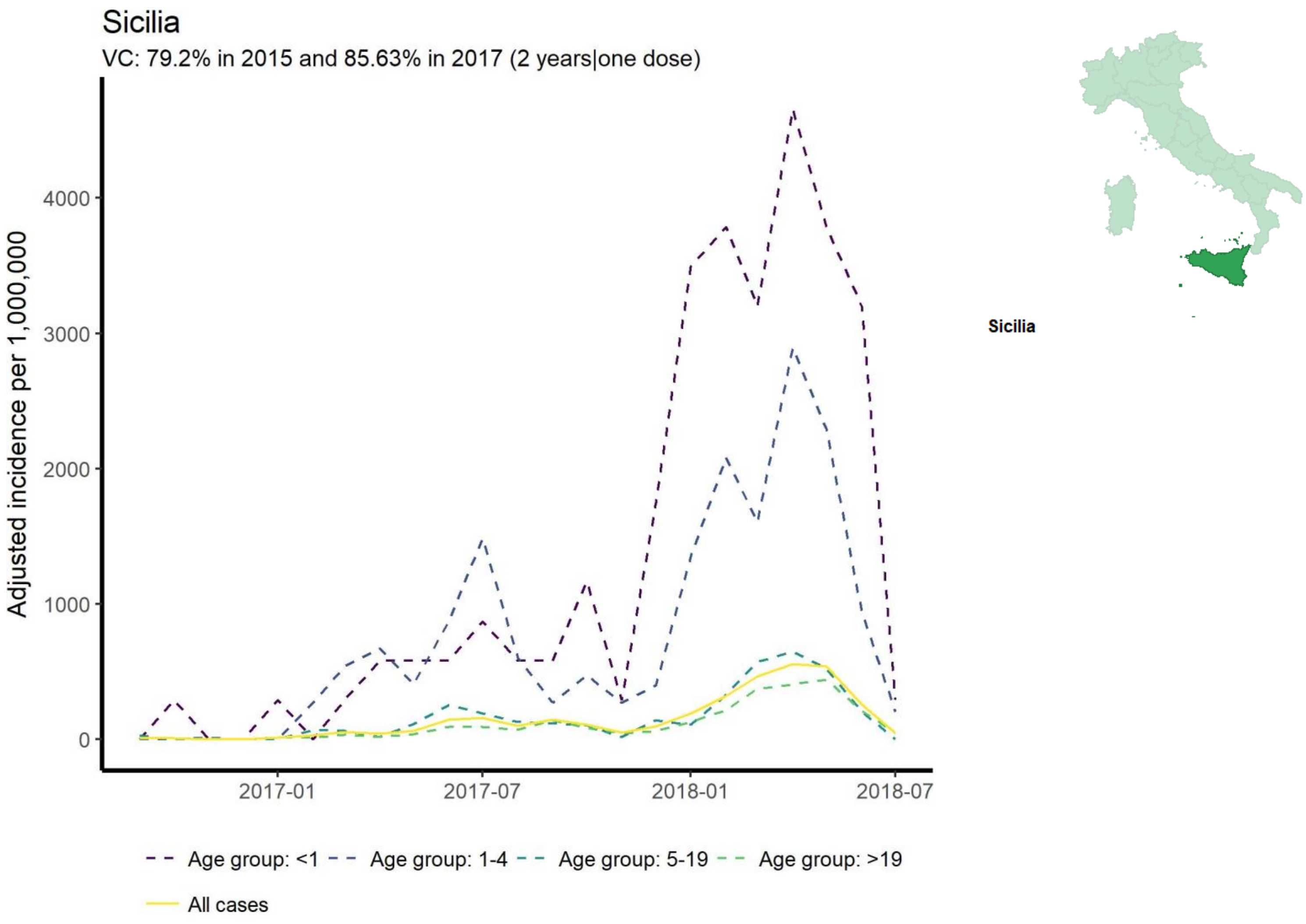

Sicilia
VC: 79.2% in 2015 and 85.63% in 2017 (2 years|one dose)
Adjusted incidence per 1,000,000
4000
3000
2000
1000
0
2017-01
2017-07
2018-01
2018-07
Age group: <1
Age group: 1-4
Age group: 5-19
Age group: >19
All cases
Sicilia

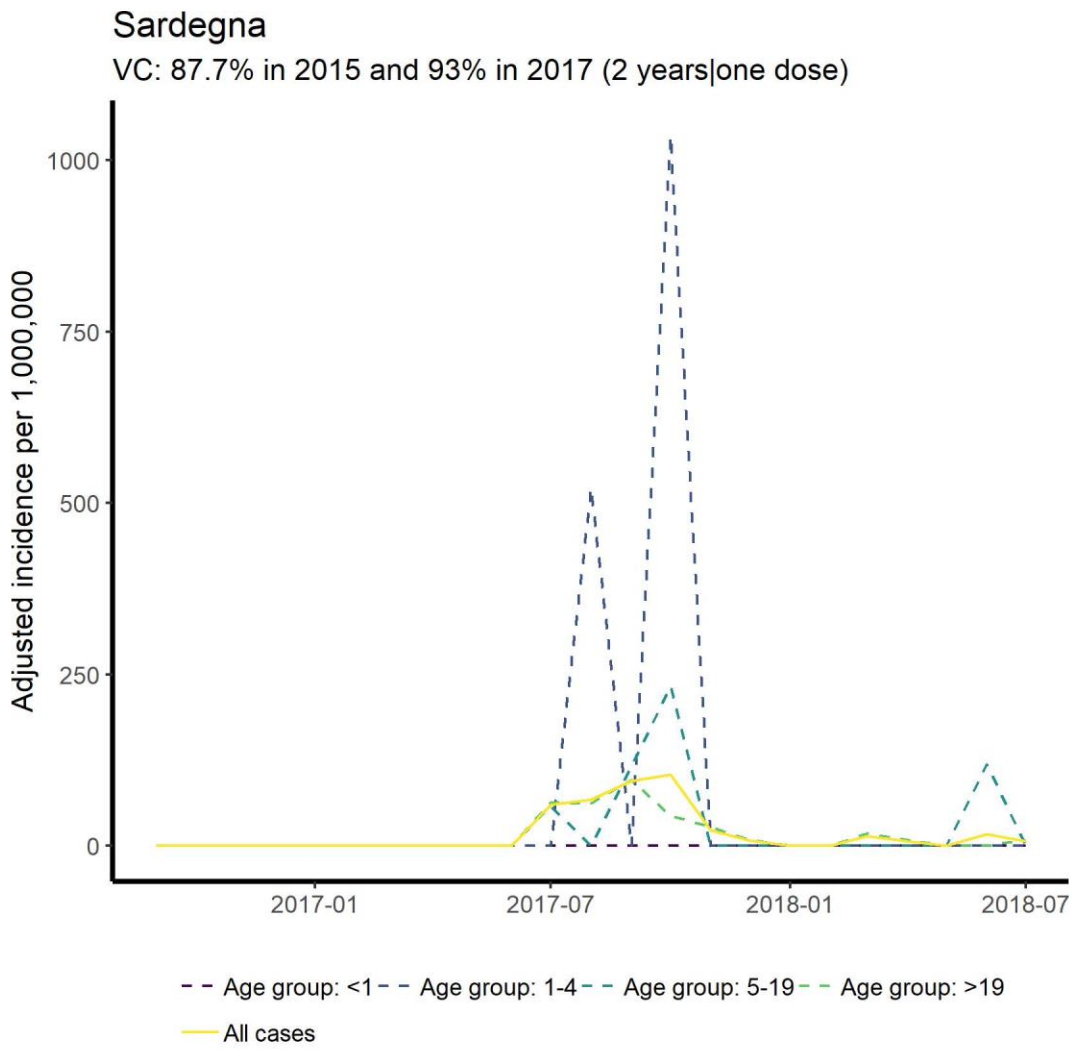
Sardegna
VC: 87.7% in 2015 and 93% in 2017 (2 years|one dose)
Adjusted incidence per 1,000,000
1000
750
500
250
0
2017-01
2017-07
2018-01
2018-07
Age group: <1
Age group: 1-4
Age group: 5-19
Age group: >19
All cases

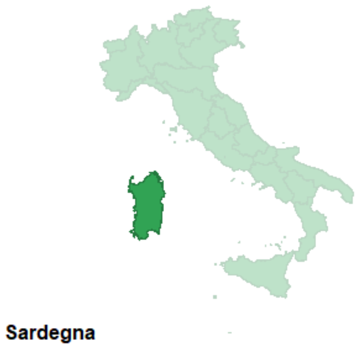
Sardegna

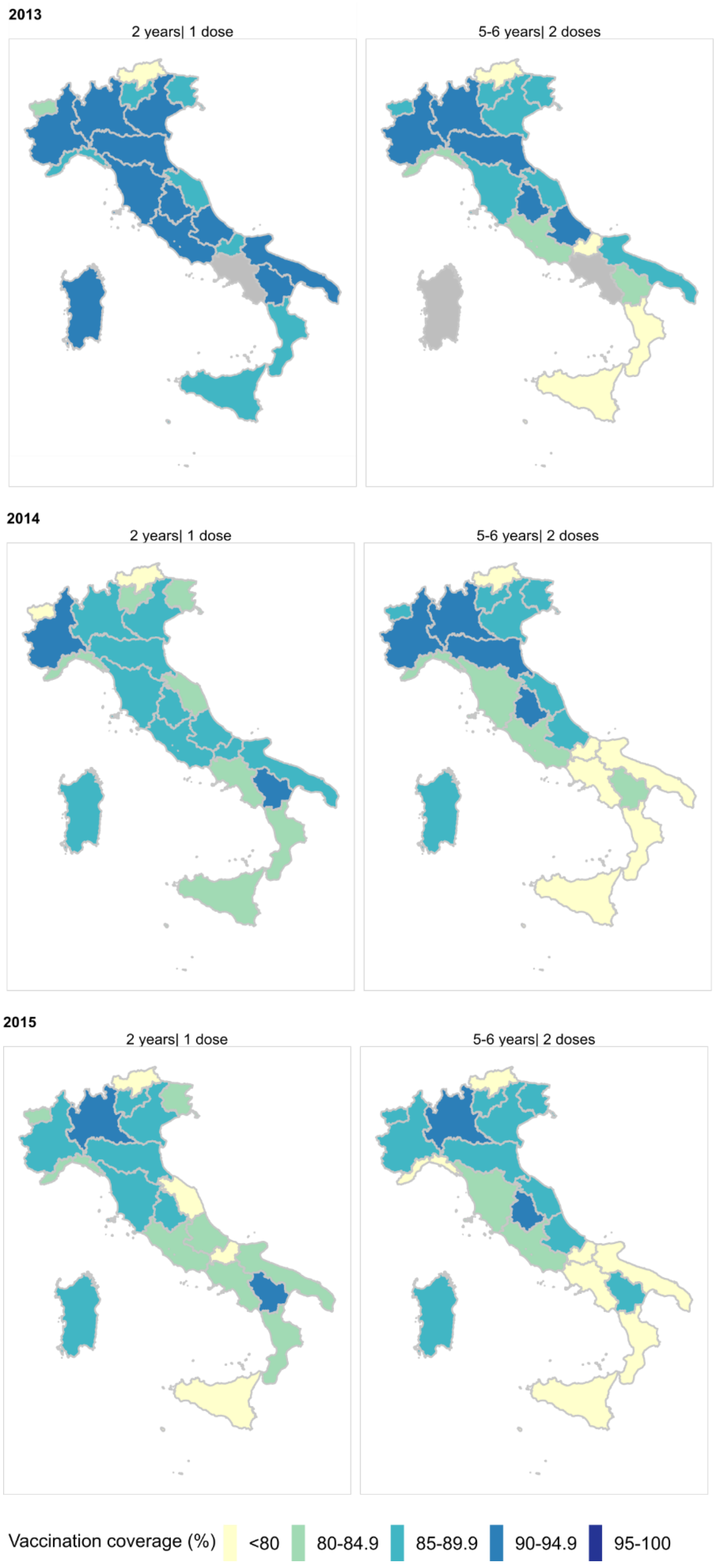
2013
2 years| 1 dose
5-6 years| 2 doses
2014
2 years| 1 dose
5-6 years| 2 doses
2015
2 years| 1 dose
5-6 years| 2 doses
Vaccination coverage (%)
<80
80-84.9
85-89.9
90-94.9
95-100

Figure S 4 Maps of the vaccine coverage in Italy, by region (2013-2015), for 2 year olds (one MMR dose), and 5-6 year olds (two MMR doses), based on the data released by the Italian Ministry of Health.

Table S 1 Reference list of the R packages used in the data analysis

1. Gohel D. rvg: R Graphics Devices for Vector Graphics Output [Internet]. 2018. Available from: https://CRAN.R-project.org/package=rvg
2. Gohel D. officer: Manipulation of Microsoft Word and PowerPoint Documents [Internet]. 2018. Available from: https://CRAN.R-project.org/package=officer
3. Wickham H. The Split-Apply-Combine Strategy for Data Analysis. Journal of Statistical Software. 2011;40(1):1–29.
4. Hlavac M. stargazer: Well-Formatted Regression and Summary Statistics Tables [Internet]. Bratislava, Slovakia: Central European Labour Studies Institute (CELSI); 2018. Available from: https://CRAN.R-project.org/package=stargazer
5. Zeileis A, Hothorn T. Diagnostic Checking in Regression Relationships. R News. 2002;2(3):7–10.
6. Varrichio C. rowr: Row-Based Functions for R Objects [Internet]. 2016. Available from: https://CRAN.R-project.org/package=rowr
7. Wickham H, Miller E. haven: Import and Export “SPSS”, “Stata” and “SAS” Files [Internet]. 2018. Available from: https://CRAN.R-project.org/package=haven
8. Wickham H. scales: Scale Functions for Visualization [Internet]. 2018. Available from: https://CRAN.R-project.org/package=scales
9. Xie Y. Dynamic Documents with R and knitr [Internet]. 2nd ed. Boca Raton, Florida: Chapman and Hall/CRC; 2015. Available from: https://yihui.name/knitr/
10. Xie Y. knitr: A Comprehensive Tool for Reproducible Research in R. In: Stodden V, Leisch F, Peng RD, editors. Implementing Reproducible Computational Research [Internet]. Chapman and Hall/CRC; 2014. Available from: http://www.crcpress.com/product/isbn/9781466561595
11. Yoshida K, Bohn J. tableone: Create “Table 1” to Describe Baseline Characteristics [Internet]. 2018. Available from: https://CRAN.R-project.org/package=tableone
12. Xie Y. knitr: A General-Purpose Package for Dynamic Report Generation in R [Internet]. 2018. Available from: https://yihui.name/knitr/
13. Bates D, Mächler M, Bolker B, Walker S. Fitting Linear Mixed-Effects Models Using lme4. Journal of Statistical Software. 2015;67(1):1–48.
14. Muller K, Wickham H. tibble: Simple Data Frames [Internet]. 2018. Available from: https://CRAN.R-project.org/package=tibble
15. Dowle M, Srinivasan A. data.table: Extension of `data.frame` [Internet]. 2018. Available from: https://CRAN.R-project.org/package=data.table
16. Aragon TJ. epitools: Epidemiology Tools [Internet]. 2017. Available from: https://CRAN.R-project.org/package=epitools
17. Wickham H. Reshaping data with the reshape package. Journal of Statistical Software [Internet]. 2007;21(12). Available from: http://www.jstatsoft.org/v21/i12/paper
18. Stabler B. shapefiles: Read and Write ESRI Shapefiles [Internet]. 2013. Available from: https://CRAN.R-project.org/package=shapefiles
19. Venables WN, Ripley BD. Modern Applied Statistics with S [Internet]. Fourth. New York: Springer; 2002. Available from: http://www.stats.ox.ac.uk/pub/MASS4
20. Hijmans RJ. raster: Geographic Data Analysis and Modeling [Internet]. 2018. Available from: https://CRAN.R-project.org/package=raster
21. Bivand R, Rundel C. rgeos: Interface to Geometry Engine - Open Source ('GEOS’) [Internet]. 2018. Available from: https://CRAN.R-project.org/package=rgeos
22. Wickham H, Hester J, Francois R. readr: Read Rectangular Text Data [Internet]. 2017. Available from: https://CRAN.R-project.org/package=readr
23. R Core Team. foreign: Read Data Stored by “Minitab”, “S”, “SAS”, “SPSS”, “Stata”, “Systat”, “Weka”, “dBase”, ... [Internet]. 2017. Available from: https://CRAN.R-project.org/package=foreign

24. Bivand RS, Pebesma E, Gomez-Rubio V. Applied spatial data analysis with R, Second edition [Internet]. Springer, NY; 2013. Available from: http://www.asdar-book.org/
25. Bivand R, Lewin-Koh N. maptools: Tools for Handling Spatial Objects [Internet]. 2018. Available from: https://CRAN.R-project.org/package=maptools
26. Bivand R, Hauke J, Kossowski T. Computing the Jacobian in Gaussian spatial autoregressive models: An illustrated comparison of available methods. Geographical Analysis. 2013;45(2):150–179.
27. Bivand R, Piras G. Comparing Implementations of Estimation Methods for Spatial Econometrics. Journal of Statistical Software. 2015;63(18):1–36.
28. Bivand R, Wong DWS. Comparing implementations of global and local indicators of spatial association. TEST. 2018;27(3):716–748.
29. Bivand RS, Pebesma E, Gomez-Rubio V. Applied spatial data analysis with R, Second edition [Internet]. Springer, NY; 2013. Available from: http://www.asdar-book.org/
30. Wickham H, Henry L. tidyr: Easily Tidy Data with “spread()” and “gather()” Functions [Internet]. 2018. Available from: https://CRAN.R-project.org/package=tidyr
31. Pebesma EJ, Bivand RS. Classes and methods for spatial data in R. R News. 2005 Nov;5(2):9–13.
32. Zeileis A, Grothendieck G. zoo: S3 Infrastructure for Regular and Irregular Time Series. Journal of Statistical Software. 2005;14(6):1–27.
33. Wickham H, Bryan J. readxl: Read Excel Files [Internet]. 2018. Available from: https://CRAN.R-project.org/package=readxl
34. Grolemund G, Wickham H. Dates and Times Made Easy with lubridate. Journal of Statistical Software. 2011;40(3):1–25.
35. Neuwirth E. RColorBrewer: ColorBrewer Palettes [Internet]. 2014. Available from: https://CRAN.R-project.org/package=RColorBrewer
36. Dowle M, Srinivasan A. data.table: Extension of `data.frame` [Internet]. 2018. Available from: https://CRAN.R-project.org/package=data.table
37. Wickham H, Fran�ois R, Henry L, M�ller K. dplyr: A Grammar of Data Manipulation [Internet]. 2018. Available from: https://CRAN.R-project.org/package=dplyr
38. Venables WN, Ripley BD. Modern Applied Statistics with S [Internet]. Fourth. New York: Springer; 2002. Available from: http://www.stats.ox.ac.uk/pub/MASS4
39. Wickham H. ggplot2: Elegant Graphics for Data Analysis [Internet]. Springer-Verlag New York; 2016. Available from: http://ggplot2.org
40. Bivand R, Lewin-Koh N. maptools: Tools for Handling Spatial Objects [Internet]. 2018. Available from: https://CRAN.R-project.org/package=maptools
41. Bivand R, Keitt T, Rowlingson B. rgdal: Bindings for the “Geospatial” Data Abstraction Library [Internet]. 2018. Available from: https://CRAN.R-project.org/package=rgdal

Table S 2 RECORD checklist* of items that need to be reported in studies of routinely collected data. The items that are unique to the STROBE statement (e.g. those referring to study design of observational studies) have been deleted from the table for brevity.

	Item No.	STROBE items	RECORD items	Location in manuscript where items are reported
**Title and abstract**				
	1	(a) Indicate the study's design with a commonly used term in the title or the abstract (b) Provide in the abstract an informative and balanced summary of what was done and what was found	RECORD 1.1: The type of data used should be specified in the title or abstract. When possible, the name of the databases used should be included.	Title
			RECORD 1.2: If applicable, the geographic region and timeframe within which the study took place should be reported in the title or abstract.	Title
			RECORD 1.3: If linkage between databases was conducted for the study, this should be clearly stated in the title or abstract.	Not applicable
**Introduction**				
Background rationale	2	Explain the scientific background and rationale for the investigation being reported		Introduction
Objectives	3	State specific objectives, including any prespecified hypotheses		Introduction
**Methods**				
Study Design	4	Present key elements of study design early in the paper		Not applicable
Setting	5	Describe the setting, locations, and relevant dates, including periods of recruitment, exposure, follow-up, and data collection		Not applicable

Participants	6	RECORD 6.1: The methods of study population selection (such as codes or algorithms used to identify subjects) should be listed in detail. If this is not possible, an explanation should be provided.<br><br>RECORD 6.2: Any validation studies of the codes or algorithms used to select the population should be referenced. If validation was conducted for this study and not published elsewhere, detailed methods and results should be provided.<br>RECORD 6.3: If the study involved linkage of databases, consider use of a flow diagram or other graphical display to demonstrate the data linkage process, including the number of individuals with linked data at each stage.		Methods and flow chart in the supplementary information<br><br>Not applicable<br><br>Not applicable
Variables	7	Clearly define all outcomes, exposures, predictors, potential confounders, and effect modifiers. Give diagnostic criteria, if applicable.	RECORD 7.1: A complete list of codes and algorithms used to classify exposures, outcomes, confounders, and effect modifiers should be provided. If these cannot be reported, an explanation should be provided.	Methods (case definitions, and additional information on case classification)
Data sources/ measurement	8	For each variable of interest, give sources of data and details of methods of assessment (measurement).<br><br>Describe comparability of assessment methods if there is more than one group		Methods (details on the source of all data included in the analysis are included)<br>Not applicable
Bias	9	Describe any efforts to address potential sources of bias		Not applicable (limitations are discussed)
Study size	10	Explain how the study size was arrived at		Not applicable
Quantitative variables	11	Explain how quantitative variables were handled in the analyses. If applicable, describe which groupings were chosen, and why		Methods
Statistical methods	12	(a) Describe all statistical methods, including those used to control for confounding		Methods

		(b) Describe any methods used to examine subgroups and interactions	Not applicable (analysis by age group is of descriptive nature)
		(c) Explain how missing data were addressed	Methods
		(e) Describe any sensitivity analyses	Methods
Data access and cleaning methods		..<br>RECORD 12.1: Authors should describe the extent to which the investigators had access to the database population used to create the study population.	Not applicable
		RECORD 12.2: Authors should provide information on the data cleaning methods used in the study.	Methods
Linkage		..<br>RECORD 12.3: State whether the study included person-level, institutional-level, or other data linkage across two or more databases. The methods of linkage and methods of linkage quality evaluation should be provided.	Not applicable
**Results**			
Participants	13	(a) Report the numbers of individuals at each stage of the study (*e.g.*, numbers potentially eligible, examined for eligibility, confirmed eligible, included in the study, completing follow-up, and analysed)<br>RECORD 13.1: Describe in detail the selection of the persons included in the study (*i.e.,* study population selection) including filtering based on data quality, data availability and linkage. The selection of included persons can be described in the text and/or by means of the study flow diagram.	Results
		(b) Give reasons for non-participation at each stage.	Methods
		(c) Consider use of a flow diagram	Flow chart included in the supplementary material
Descriptive data	14	(a) Give characteristics of study participants (*e.g.*, demographic, clinical, social) and information on exposures and potential confounders	Table 1

		(b) Indicate the number of participants with missing data for each variable of interest		Methods and Table 1
Main results	16	(a) Give unadjusted estimates and, if applicable, confounder-adjusted estimates and their precision (e.g., 95% confidence interval). Make clear which confounders were adjusted for and why they were included		Results (the estimates and 95% Cis of the regression analysis results are provided)
		(b) Report category boundaries when continuous variables were categorized		Reported in Table 2 and the results
		(c) If relevant, consider translating estimates of relative risk into absolute risk for a meaningful time period		All incidence rates have been translated to rates per 1,000,000 per year for comparability
Other analyses	17	Report other analyses done—e.g., analyses of subgroups and interactions, and sensitivity analyses		Sensitivity analysis results mentioned
**Discussion**				
Key results	18	Summarise key results with reference to study objectives		Beginning of the discussion
Limitations	19	Discuss limitations of the study, taking into account sources of potential bias or imprecision. Discuss both direction and magnitude of any potential bias	RECORD 19.1: Discuss the implications of using data that were not created or collected to answer the specific research question(s). Include discussion of misclassification bias, unmeasured confounding, missing data, and changing eligibility over time, as they pertain to the study being reported.	Discussion
Interpretation	20	Give a cautious overall interpretation of results considering objectives, limitations, multiplicity of analyses, results from similar studies, and other relevant evidence		Discussion

Generalisability	21	Discuss the generalisability (external validity) of the study results	Discussion
**Other Information**			
Funding	22	Give the source of funding and the role of the funders for the present study and, if applicable, for the original study on which the present article is based	Not applicable
Accessibility of protocol, raw data, and programming code		.. RECORD 22.1: Authors should provide information on how to access any supplemental information such as the study protocol, raw data, or programming code.	Addressed in the methods

*Reference: Benchimol EI, Smeeth L, Guttmann A, Harron K, Moher D, Petersen I, Sørensen HT, von Elm E, Langan SM, the RECORD Working Committee. The REporting of studies Conducted using Observational Routinely-collected health Data (RECORD) Statement. *PLoS Medicine* 2015; in press.

*Checklist is protected under Creative Commons Attribution (CC BY) license.

Table S 3 Incidence (per 1,000,000 per year) by age group for Italy and the regions of Lazio and Sicily, September 2016-July 2017.

Age group	Italy	Lazio*	Sicily*
**<1**	529.9	1550.2	1316.5
**1-4**	251.4	548.3	766.7
**5-19**	66.5	174.3	158.5
**>19**	55.9	168.8	110.9
***Age-adjusted using the Italian population (2017) as a reference.**			

Table S 4 Summary statistics of vaccine coverage (%) for the first dose of MMR vaccine at 2 years of age, by region, between 2000 and 2017.

Region	Mean	Min	Median	Max
**Abruzzo**	88.9	76.7	89.2	93.1
**Basilicata**	87.9	58.9	90.1	96.6
**Calabria**	81.0	53.4	84.8	92.8
**Campania**	82.1	53.0	84.4	92.0
**Emilia-Romagna**	91.8	87.1	92.7	93.9
**Friuli-Venezia Giulia**	89.6	81.9	91.0	92.9
**Lazio**	86.7	66.8	89.0	95.3
**Liguria**	83.9	69.3	86.2	90.9
**Lombardia**	91.4	81.5	92.8	94.9
**Marche**	87.9	79.9	88.0	96.7
**Molise**	83.8	59.9	86.9	97.3
**Piemonte**	87.5	67.6	90.2	94.7
**Provincia Autonoma di Bolzano**	65.6	47.7	67.7	75.9
**Provincia Autonoma di Trento**	84.7	70.0	86.8	91.7
**Puglia**	88.9	77.3	90.2	93.4
**Sardegna**	90.4	84.2	90.5	95.8
**Sicilia**	84.8	79.2	84.8	90.1
**Toscana**	88.9	77.6	89.8	93.5
**Umbria**	91.4	84.4	93.0	95.4
**Valle d'Aosta**	82.9	56.0	86.9	91.8
**Veneto**	90.9	87.1	91.7	93.0

Table S 5 Regions with at least one year of VC 95% or above at 2 years (between 2000 and 2017).

Year	Birth Cohort	Region	%VC (2 years old|1 dose)
**2003**	2001	Basilicata	**96.6**
**2012**	2010	Basilicata	**95.0**
**2017**	2015	Lazio	**95.3**
**2012**	2010	Marche	**96.7**
**2007**	2005	Molise	**97.3**
**2009**	2007	Sardegna	**95.5**
**2011**	2009	Sardegna	**95.8**
**2009**	2007	Umbria	**95.2**
**2010**	2008	Umbria	**95.4**

Table S 6 Regression analysis results (univariable and multivariable) without adjustment for the regions.

		Univariable models				Multivariable model			
		**IRR**	**2.5% CI**	**97.5% CI**	**p-value**	**IRR**	**2.5% CI**	**97.5% CI**	**p-value**
**Degree of urbanization Reference category: Thinly populated areas**	**Densely populated areas**	1.249	0.989	1.594	0.071	1.171	0.924	1.500	0.208
	**Intermediate**	1.206	1.071	1.360	0.002	1.101	0.969	1.251	0.141
**Social deprivation index category Reference category: SD 5 (most deprived)**	**SD 1 (least deprived)**	0.772	0.641	0.929	0.007	0.737	0.609	0.890	0.002
	**SD 2**	0.745	0.621	0.894	0.002	0.725	0.601	0.874	0.000
	**SD 3**	0.742	0.617	0.892	0.002	0.755	0.624	0.914	0.004
	**SD 4**	1.091	0.918	1.295	0.335	1.111	0.929	1.328	0.257
**Percentage of adults (≥18 years old) Reference category: >86.65**	**[73.14, 82.76]**	1.782	1.441	2.215	<0.001	1.720	1.370	2.167	<0.001
	**(82.76, 84.64]**	1.959	1.581	2.438	<0.001	1.935	1.549	2.426	<0.001
	**(84.64, 86.65]**	1.604	1.285	2.012	<0.001	1.604	1.279	2.018	<0.001

Table S 7 Multivariable analysis results including only the municipalities with a population below 50,000 in 2017.

		Multivariable model (adjusted for the region as a random effect)			
		IRR	2.5% CI	97.5% CI	p-value
Degree of urbanization Reference category: Thinly populated areas	Densely populated areas	1.512	1.113	2.055	0.008
	Intermediate	1.162	1.021	1.322	0.023
Social deprivation index category Reference category: SD 5 (most deprived)	SD 1 (least deprived)	1.350	1.000	1.821	0.050
	SD 2	1.095	0.821	1.461	0.537
	SD 3	0.913	0.701	1.189	0.499
	SD 4	1.058	0.862	1.298	0.590
Percentage of adults (≥18 years old) Reference category: >86.65	[73.14, 82.76]	1.845	1.457	2.335	<0.001
	(82.76, 84.64]	1.915	1.529	2.398	<0.001
	(84.64, 86.65]	1.560	1.244	1.956	<0.001
